# Screening in High Schools to Identify, Evaluate, and Lower Depression Among Adolescents: A Randomized Clinical Trial

**DOI:** 10.1001/jamanetworkopen.2021.31836

**Published:** 2021-11-05

**Authors:** Deepa L. Sekhar, Eric W. Schaefer, James G. Waxmonsky, Leslie R. Walker-Harding, Krista L. Pattison, Alissa Molinari, Perri Rosen, Jennifer L. Kraschnewski

**Affiliations:** 1 Department of Pediatrics, Pennsylvania State College of Medicine, Hershey; 2 Department of Public Health Sciences, Pennsylvania State College of Medicine, Hershey; 3 Department of Psychiatry, Pennsylvania State College of Medicine, Hershey; 4 Department of Pediatrics, Seattle Children’s, Seattle, Washington; 5 Statewide Project Advisor, Garrett Lee Smith Youth Suicide Prevention Grant, Harrisburg, Pennsylvania; 6 Department of Medicine, Pennsylvania State College of Medicine, Hershey

## Abstract

**Importance:**

Adolescent major depressive disorder (MDD) prevalence has nearly doubled in the past decade. The US Preventive Services Task Force endorses universal adolescent MDD screening in primary care; however, most adolescents lack preventive health care, resulting in worsening disparities in MDD screening and treatment.

**Objective:**

To evaluate the effectiveness of universal adolescent MDD screening in the school setting in an effort to reduce disparities and improve MDD identification and treatment initiation.

**Design, Setting, and Participants:**

This randomized clinical trial, conducted from November 6, 2018, to November 20, 2020, compared the usual school practice of targeted or selected screening based on observable behaviors of concern with universal MDD screening. Students within an identified school were randomized by grade to 1 of the 2 study groups. Study groups were compared using mixed-effects logistic regression. Participants included students in grades 9 through 12 enrolled at 1 of the 14 participating Pennsylvania public high schools.

**Interventions:**

In targeted screening, students with behaviors prompting concern for MDD were referred to the Student Assistance Program (SAP), mandated in all Pennsylvania schools. The SAP determined follow-up recommendations. In universal screening, all students completed the Patient Health Questionnaire-9 (PHQ-9); students with positive scores proceeded to SAP. The universal screening group could also have targeted referral to SAP for concerning behavior independent of the PHQ-9.

**Main Outcomes and Measures:**

The primary outcome was initiation of MDD treatment or services based on data collected by school SAP teams during the academic year.

**Results:**

A total of 12 909 students were included (median age, 16 years [range, 13-21 years]; 6963 male [53.9%]), of whom 2687 (20.8%) were Hispanic, 2891 (22.4%) were non-Hispanic Black, 5842 (45.3%) were non-Hispanic White, and 1489 (11.5%) were multiracial or of other race or ethnicity. A total of 6473 students (50.1%) were randomized to universal screening, and 6436 (49.9%) were randomized to targeted screening. Adolescents in the universal screening group had 5.92 times higher odds (95% CI, 5.07-6.93) of being identified with MDD symptoms, 3.30 times higher odds (95% CI, 2.49-4.38) of SAP confirming follow-up needs, and 2.07 times higher odds (95% CI, 1.39-3.10) of initiating MDD treatment. No differences were identified in initiation for planned subgroup analyses by sex or race and ethnicity.

**Conclusions and Relevance:**

In this randomized clinical trial, universal school-based MDD screening successfully increased identification of MDD symptoms and treatment initiation among adolescents, confirming the value of this approach to address this rising public health concern.

**Trial Registration:**

ClinicalTrials.gov identifier: NCT03716869

## Key Points

**Question:**

Can universal adolescent major depressive disorder (MDD) screening conducted in the school setting improve MDD identification and treatment initiation compared with targeted student referral based on observable behaviors of concern?

**Findings:**

In a randomized clinical trial including 12 909 students attending 14 Pennsylvania public high schools, adolescents randomized to universal screening vs the usual process of targeted student referral had significantly higher odds both of being identified with MDD symptoms (5.9 times higher) and of initiating MDD treatment (2.1 times higher).

**Meaning:**

In this trial, universal school-based MDD screening successfully increased treatment initiation among identified students.

## Teaser

This randomized clinical trial investigates whether universal screening for major depressive disorder among adolescents improves identification and treatment of the disorder vs targeted screening of observable behaviors of concern.

## Introduction

The prevalence of adolescents reporting major depressive disorder (MDD) symptoms has nearly doubled in the last decade, increasing from 8.3% in 2008 to 14.4% in 2018.^1^ To better screen for and identify MDD, the US Preventive Services Task Force (USPSTF) endorsed primary care screening in 2009 for all adolescents aged 12 to 18 years in settings with capacity to ensure appropriate follow-up, reaffirming this statement in 2016.^2^ The USPSTF review supported that screening instruments can accurately identify adolescent MDD and that treatment of adolescents with screen-detected MDD is associated with beneficial reductions in symptoms.^2^

Unfortunately, MDD screening has significant disparities.^3,4^ Most US adolescents (>60%) lack routine preventive health care, limiting the ability of primary care to address this worsening public health concern.^5,6^ Furthermore, primary care MDD screening is inconsistent, with inequalities by race and ethnicity and region, and potential worsening with the COVID-19 pandemic.^3,4,7^ Additional gaps identified by the USPSTF include the need for large randomized clinical trials (RCTs) to understand the effects of MDD screening and to quantify the proportion of individuals with screen-detected MDD successfully treated.^2^

Innovative approaches are needed to improve MDD screening and treatment. Schools create a more equitable setting for screening given that most adolescents attend, regardless of race, ethnicity, and socioeconomic status. Thus, we identified schools as an ideal setting to (1) evaluate universal adolescent MDD symptom screening, (2) compare the effectiveness of universal vs targeted MDD symptom screening, and (3) address disparities in MDD screening.^8^ Prior studies of school-based MDD screening demonstrate increased identification and referral, but limited data exist on treatment initiation.^9,10,11,12^ An RCT by Huskey et al^13^ of 4 Pennsylvania high schools demonstrated increased treatment initiation with screening. An RCT of depression screening by Guo et al^14^ in California middle schools found no effect of universal screening on treatment initiation.

Adolescents’ consistent contact with schools has been used to support physical health screenings that affect academic success.^15^ Major depressive disorder similarly affects academic success, suggesting school-based screening may be especially beneficial.^2,16,17^ This article reports results of the Screening in High Schools to Identify, Evaluate, and Lower Depression (SHIELD) RCT, conducted in Pennsylvania public high schools from November 6, 2018, to November 20, 2020, to compare the effectiveness of universal school-based MDD screening vs targeted screening on MDD treatment initiation. Our hypothesis was that universal screening would increase treatment initiation among identified students and for specific subgroups: female students, adolescents from rural areas, and minority students (ie, students in racial and ethnic minority groups).

## Methods

### Trial Design

The SHIELD protocol (Supplement 1) was approved by the Pennsylvania State University College of Medicine Institutional Review Board and previously published.^8^ Schools were recruited leveraging prior research team relationships.^8,18,19^ Schools were identified for recruitment to ensure a statewide sample of rural and urban schools with diverse student demographic backgrounds as outlined by the funding agencies (based on data from the Pennsylvania school performance profile^20^) and interest in participation. Recruitment telephone calls and emails went to 45 high schools on these criteria to initiate conversations with school personnel; 11 never responded. Contact and initial responses were received from 34 high schools, and 16 initially enrolled. However, 1 school underwent leadership changes and declined participation after randomization but before screening. Another declined participation after the first day of screening 82 students (estimated 3% of enrollment), citing demands on staff time, and did not provide further data.

Schools were randomized to either (1) 9th and 11th graders assigned to universal screening, or (2) 10th and 12th graders assigned to universal screening, with the other grades assigned to targeted screening.^8^ School students and staff were not blinded to study group. All 9th- to 12th-grade students were eligible for participation aside from those whose parents opted out or students with disabilities deemed unable to participate by the school. Letters sent to parents or guardians before screening detailed the project and screening instrument, the Patient Health Questionnaire-9 (PHQ-9), and offered the opportunity to opt their student out of the study.^8,21^ Students in the universal screening group either gave their assent by completion of the PHQ-9 or their refusal by declining to complete it at the time of screening.

Targeted screening followed current school practice. If a student exhibited behaviors raising consideration for MDD, a referral was made to the Student Assistance Program (SAP). In Pennsylvania public schools, SAP is the primary process to address the needs of students who display any barrier to learning.^22^ The SAP is a professionally trained team including school staff and liaisons from mental health agencies. The SAP uses a team process to gather and review data on observable behaviors or symptoms prompting the referral. The SAP does not diagnose, but after review, students confirmed to require intervention are referred for follow-up school-based and/or community-based services, which may be based on screening or assessment by a SAP liaison from a community behavioral health agency.^22^

Students in universal screening were administered a PHQ-9 electronically (via 10 study-purchased iPads [Apple Inc]) during school hours by the study team. This screening occurred in classrooms (eg, all ninth-grade English classes) or in a designated space, eg, school library or auditorium, with direct entry into REDCap (Research Electronic Data Capture), a secure, web-based application designed to support research studies.^23^ The PHQ-9 is a well-established, validated screening tool; a score greater than 10 has a sensitivity of 89.5%, a specificity of 77.5%, and a positive predictive value of 15.2% for the diagnosis of MDD among adolescents.^21^ Students with a positive result (PHQ-9 score >10) were referred to SAP using the same process as in the targeted screening arm. Those with suicidality, based on a score of 1 or greater for PHQ-9 item No. 9, were reported to designated school staff on the day of screening regardless of total score. Students in the universal screening group who exhibited behaviors concerning for MDD any time during the academic year were referred to SAP via the same process as for the targeted screening group.

Three schools participated in the RCT in the 2018-2019 academic year and 11 in the 2019-2020 academic year. The COVID-19 pandemic resulted in Pennsylvania school closure on March 15, 2020,^24^ affecting screening completion at 2 schools. The SAP team functions were variably affected; online meeting platforms and telehealth were used to support continued services.^25^ This study followed the Consolidated Standards of Reporting Trials (CONSORT) reporting guideline.

### Outcome Measures

The primary outcome was the percentage of students confirmed by SAP with MDD symptoms that warranted further evaluation who initiated recommended treatment. To meet the primary outcome including definitions for MDD symptoms and treatment, students were required to meet all of the following criteria: (1) identified (MDD symptoms): positive PHQ-9 (score >10 or response ≥1 on question 9) or behavior prompting referral to SAP for MDD; (2) confirmed: SAP does not diagnose, but to meet criteria, SAP must confirm that identified behavior or symptoms warrant further evaluation; (3) initiated: participated in at least 1 SAP-recommended treatment or service for MDD, eg, follow-up mental health supports. Confirmation by school staff (criteria 2) is similar to implementation as described in the USPSTF statement, whereby initial screening is followed by a second phase in which the student’s individual situation is considered in making further recommendations.^2,22^ Students already in treatment did not meet criteria for initiation. If students accidentally completed the PHQ-9 more than once because of school error, a student was identified based on a positive score on any of these completions. Additional details about how the criteria were operationalized are described in eTable 1 in Supplement 2. Secondary outcomes were the first and second criteria (identified and confirmed).

### Statistical Analysis

Based on Pennsylvania SAP data, we planned to enroll at least 8 schools (n = 9650 students), which yielded greater than 99% power to detect a difference of 5% vs 2% in the universal and targeted screening groups, respectively, for the primary outcome.^26^ The sample size had greater than 80% power for planned subgroup analyses by sex and race and ethnicity to understand the effectiveness of screening on disparities in MDD identification and treatment initiation.^8^ Demographic data were based on school records.

Statistical analysis was conducted using the intent-to-treat principle. The analysis for primary and secondary outcomes that compared universal to targeted screening was conducted using mixed-effects logistic regression.^27^ Models contained a fixed effect for randomized group and a random effect for school. Odds ratios (ORs) and 95% CIs were reported. For the planned subgroup analyses of sex, race and ethnicity, and school location, the same models were used but were extended by adding fixed effects for subgroup and interaction effects for subgroup by randomized group. We reported the *P* value for the joint test of all interaction terms and reported ORs (95% CIs) for the interactions when the joint test was significant (*P* < .05). All *P* values were 2-sided. Statistical analyses were performed using R software, version 4.0.2 (R Foundation) and SAS, version 9.4 (SAS Institute Inc).

Under the intent-to-treat principle, all randomized students were included in the analysis. Two students with a missing value for the primary outcome were imputed as not receiving services for the analysis. Students who withdrew or unenrolled from school might be considered to have a missing outcome because they were followed up for only part of the school year. We retained these students in the primary analysis but conducted 2 sensitivity analyses. In the first sensitivity analysis, we excluded these students entirely, which is appropriate under an assumption of missing data completely at random.^28^ In the second sensitivity analysis, we included students who unenrolled but added demographic variables to the model, which is appropriate under an assumption of missing at random.^29^

## Results

### Participants

Among 13 171 eligible students, 262 (2.0%) opted out; thus, 12 909 students were included in the analysis (Figure). A total of 5946 students (46.1%) were female and 6963 (53.9%) were male; 2687 (20.8%) were Hispanic, 2891 (22.4%) were non-Hispanic Black, 5842 (45.3%) were non-Hispanic White, and 1489 (11.5%) were multiracial or of other race or ethnicity (includes American Indian/Alaska Native, Asian, Native Hawaiian, Other Pacific Islander, and other, as some schools simply reported “other”). Median age was 16 years (range, 13-21 years). In total, 6436 students (49.9%) were randomized to targeted screening and 6473 (50.1%) to universal screening, with demographic details comparable between study groups (Table 1). Statistical analysis was based on students from 14 schools, 7 of which were classified as urban, with a median size of 370 students (range, 67-3496 students) (Table 2). Eight schools required multiple days to complete universal screening, ranging from 2 to 10 days, primarily owing to their larger student body size. Schools that required multiple days screened a median of 112 students in a given day (IQR, 91-148 students). Students completed the PHQ-9 in a median of 71 seconds (IQR, 54-95 seconds).

**Figure.**
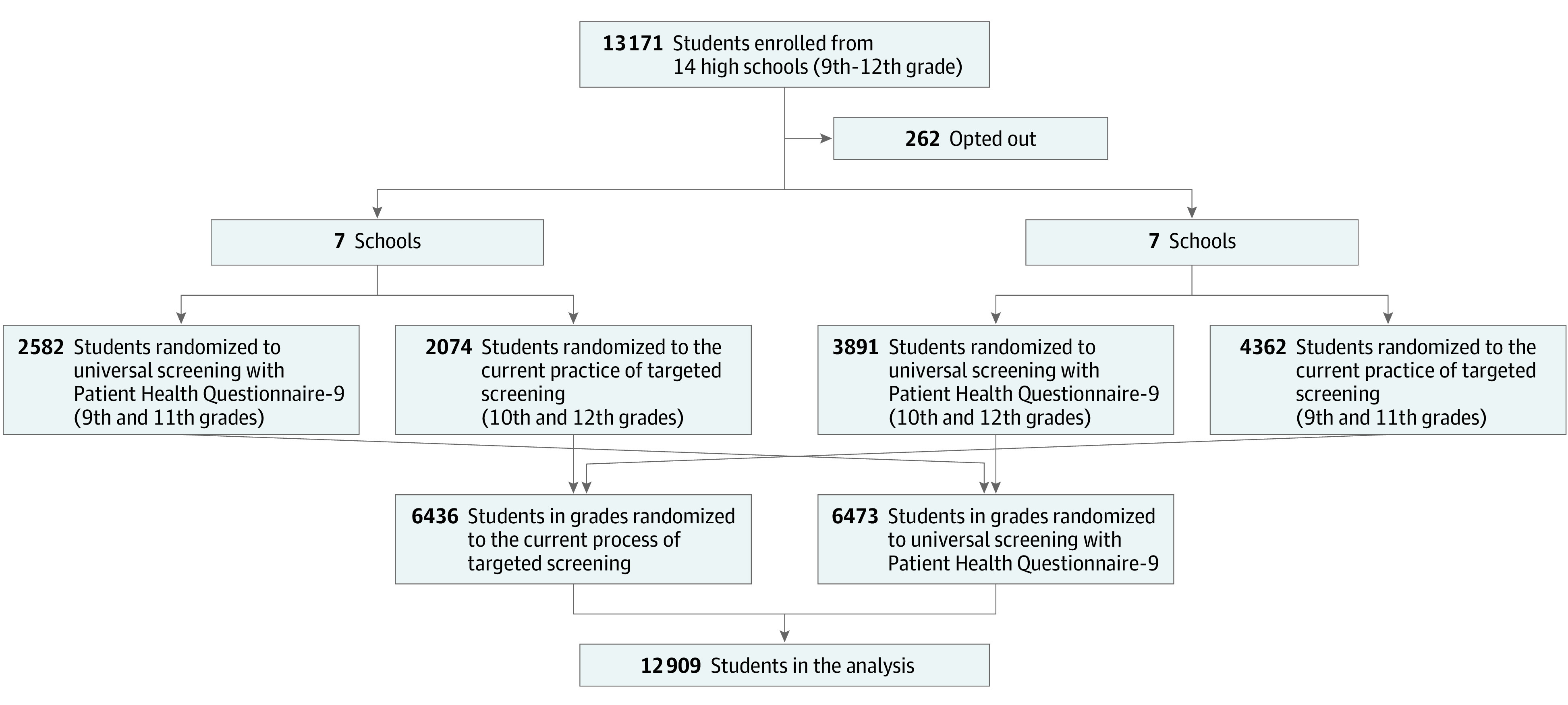
Study Flow Diagram

**Table 1.** Student Demographics and Screening by Randomized Group

Variable	No. (%)	
	Targeted screening (n = 6436)	Universal screening (n = 6473)
Grade		
9th	2430 (37.8)	1531 (23.7)
10th	1048 (16.3)	2106 (32.5)
11th	1932 (30.0)	1051 (16.2)
12th	1026 (15.9)	1785 (27.6)
Age, median (IQR), y	16 (15-17)	16 (15-17)
Female	2941 (45.7)	3005 (46.4)
Male	3495 (54.3)	3468 (53.6)
Race and ethnicity		
Hispanic (any race)	1266 (19.7)	1421 (22.0)
Non-Hispanic Black	1571 (24.4)	1320 (20.4)
Non-Hispanic White	2857 (44.4)	2985 (46.1)
Other^a^ or multiple races	742 (11.5)	747 (11.5)
School location		
Urban	5728 (89.0)	5735 (88.6)
Rural	708 (11.0)	738 (11.4)
Unenrolled during school year	121 (1.9)	216 (3.3)
Screening		
Offered or received PHQ-9		
Not offered	5977 (92.9)	1941 (30.0)
Offered but did not assent	19 (0.3)	153 (2.4)
Offered and assented	440 (6.8)	4379 (67.7)
Reasons for not being offered PHQ-9 (n = 1941)		
COVID-19 shutdown	NA	883 (45.5)
Absent (any reason) or tardy	NA	653 (33.6)
Other/no reason given	NA	405 (20.9)

Abbreviations: NA, not applicable; PHQ-9, Patient Health Questionnaire-9.

^a^ Other includes American Indian/Alaska Native, Asian, Native Hawaiian, Other Pacific Islander, and other (some schools simply reported “other”).

**Table 2.** Randomization by School and Number of Student Opt-Outs

School	Grades randomized to universal screening	Total No. of students	No. (%) of opt-outs	No. of students		
				Included in analysis	In targeted screening	In universal screening
A	10th and 12th	3496	24 (0.7)	3472	1906	1566
B	10th and 12th	2237	38 (1.7)	2199	1115	1084
C	9th and 11th	2148	3 (0.1)	2145	875	1270
D	10th and 12th	1519	13 (0.9)	1506	802	704
E	9th and 11th	1226	83 (6.8)	1143	564	579
F	10th and 12th	675	7 (1.0)	668	315	353
G	9th and 11th	380	0	380	163	217
H	9th and 11th	364	8 (2.2)	356	165	191
I	9th and 11th	336	6 (1.8)	330	151	179
J	9th and 11th	267	5 (1.9)	262	139	123
K	10th and 12th	235	17 (7.2)	218	111	107
L	10th and 12th	152	23 (15.1)	129	79	50
M	10th and 12th	69	8 (11.6)	61	34	27
N	9th and 11th	67	27 (40.3)	40	17	23
Overall	All grades	13 171	262 (2.0)	12 909	6436	6473

Among students in the universal screening group, 1941 (30.0%) did not receive the PHQ-9. The main reasons included COVID-19–related school closure for 883 students (45.5%) and absences or tardiness for 653 students (33.6%). Among students in the targeted screening arm, 459 (7.1%) were erroneously offered PHQ-9 screening. This often occurred when screening was done over multiple days, because of tracking errors or grade confusion (students between grades owing to failing classes or missing credits may have been confused on their actual grade), or when students in a grade assigned to the targeted group were in a class filled primarily with students in the universal group. A total of 172 students (1.3%) did not assent to the PHQ-9 on the screening day, and 36 students (0.3%) completed the PHQ-9 twice (on separate days).

### Primary Outcome

A total of 1226 students (9.5%) met our criteria for identification of MDD symptoms, including 1026 (15.9%) in the universal screening group and 200 (3.1%) in the targeted screening group. Students confirmed to have MDD symptoms warranting follow-up included 233 (3.6%) in the universal screening group and 64 (1.0%) in the targeted screening group, and students meeting the primary outcome of treatment initiation included 80 (1.2%) in the universal screening group and 35 (0.5%) in the targeted screening group. Based on the fitted regression models, students in the universal screening group had 5.9 times higher odds (95% CI, 5.1-6.9; *P* < .001) of being identified with MDD symptoms, 3.3 times higher odds (95% CI, 2.5-4.4; *P* < .001) of being identified and confirmed as having MDD symptoms warranting follow-up, and 2.1 times higher odds (95% CI, 1.4-3.1; *P* < .001) of initiating recommended treatment or services for MDD compared with students in the targeted screening group (Table 3).

**Table 3.** Mixed-Effects Logistic Regression Model for the Primary Analysis Evaluating Odds of Identified, Confirmed, and Initiated for Students in the Universal vs Targeted Screening Group^a^

Parameter	OR (95% CI)	*P* value	Variance of random school effect (SE)
**Identified**			
Universal screening	5.92 (5.07-6.93)	<.001	0.05 (0.04)
Targeted screening	1 [Reference]		
**Confirmed**			
Universal screening	3.30 (2.49-4.38)	<.001	1.67 (0.74)
Targeted screening	1 [Reference]		
**Initiated**			
Universal screening	2.07 (1.39-3.10)	<.001	1.60 (0.95)
Targeted screening	1 [Reference]		

Abbreviation: OR, odds ratio.

^a^ Identified major depressive disorder symptoms: positive Patient Health Questionnaire-9 (score >10 or response ≥1 on question 9) or behavior prompting referral to the Student Assistance Program for major depressive disorder; confirmed: Student Assistance Program does not diagnose, but to meet criteria, Student Assistance Program must confirm identified behavior or symptoms warrant further evaluation; initiated: participated in at least 1 Student Assistance Program–recommended treatment or service for major depressive disorder, eg, follow-up mental health supports.

Of 1226 students who screened positive, 929 (75.8%) lacked SAP confirmation that referral was warranted. In more than 80% of cases, although direct referral to outside services or meetings with school staff were recorded, no further information was documented by the school SAP, so students did not meet criteria for the primary outcome (eTable 2 in Supplement 2). Among 297 students confirmed with MDD symptoms in need of follow-up, 180 (60.6%) failed to engage in services or treatment. The primary reasons were inability to obtain parental permission (81 [45.0%]), the student already being in treatment (61 [33.9%]), and student refusal (20 [11.1%]) (eTable 3 in Supplement 2).

A total of 337 students unenrolled during the school year, 121 (1.9%) from the targeted screening group and 216 (3.3%) from the universal screening group. Students who unenrolled were more likely to be male, non-Hispanic Black, in urban schools, and in the universal screening group. A sensitivity analysis excluding students who unenrolled during the school year did not appreciably change the estimates, nor did a sensitivity analysis in which demographic variables were added to the model.

### Secondary Analysis

Three planned subgroup analyses were conducted: sex, race and ethnicity, and urban vs rural school location (Table 4; eTable 4 in Supplement 2). Interaction terms for sex by randomized group were significant for increased identification (OR, 8.0; 95% CI, 6.7-10.6 for girls; OR, 4.0; 95% CI, 3.3-5.0 for boys, *P* < .001) and confirmation (OR, 4.7; 95% CI, 3.2-7.0 for girls; OR, 2.1; 95% CI, 1.4-3.2 for boys; *P* = .005) of MDD symptoms for universal screening, with larger effects for female students, but the interaction was not significant for initiation. Similarly, interaction terms for race and ethnicity by randomized group were significant for increased identification (OR, 7.4; 95% CI, 5.0-11.2 for Hispanic students; OR, 2.6; 95% CI, 2.0-3.3 for non-Hispanic Black students; OR, 8.6; 95% CI, 6.6-11.4 for non-Hispanic White students; OR, 12.4; 95% CI, 7.3-21.0 for other race and ethnicity; *P* < .001) and confirmation (OR, 10.2; 95% CI, 4.1-25.4 for Hispanic students; OR, 4.2; 95% CI, 2.0-8.7 for non-Hispanic Black students; OR, 2.2; 95% CI, 1.6-3.2 for non-Hispanic White students; OR, 12.2; 95% CI, 1.6-94.1 for other race and ethnicity; *P* = .007) for universal screening, with the largest effects among students identified as Hispanic or other race or ethnicity, but the interaction was not significant for initiation. Finally, for school location by randomized group, interaction terms were significant for increased identification (OR, 13.6; 95% CI, 7.3-25.4 for rural location; OR, 5.5; 95% CI, 4.7-6.4 for urban location; *P* = .006) for universal screening, with larger effects for students from rural areas, but the interaction was not significant for confirmation nor for initiation.

**Table 4.** Tests of Interaction Terms From Mixed-Effects Logistic Regression Models for the Subgroup Analyses Evaluating Odds of Identified, Confirmed, and Initiated for Students in the Universal vs Targeted Screening Group^a^

Outcome	Parameter	OR (95% CI)	*P* value	Variance of school random effect (SE)
**Sex**				
Identified	Sex × rand group (interaction)	NA	<.001	0.06 (0.03)
	Male: US vs TS	4.05 (3.26-5.03)	<.001	
	Female: US vs TS	8.42 (6.71-10.58)	<.001	
Confirmed	Sex × rand group (interaction)		.005	1.70 (0.76)
	Male: US vs TS	2.09 (1.38-3.16)	<.001	
	Female: US vs TS	4.73 (3.19-7.02)	<.001	
Initiated	Sex × rand group (interaction)	NA	.37	1.61 (0.95)
**Race and ethnicity**				
Identified	Race/ethnicity × rand group (interaction)	NA	<.001	0.05 (0.03)
	Hispanic: US vs TS	7.45 (4.98-11.17)	<.001	
	Non-Hispanic Black: US vs TS	2.55 (1.97-3.31)	<.001	
	Non-Hispanic White: US vs TS	8.65 (6.58-11.35)	<.001	
	Other: US vs TS	12.41 (7.34-21.00)	<.001	
Confirmed	Race/ethnicity × rand group (interaction)	NA	.007	1.72 (0.77)
	Hispanic: US vs TS	10.15 (4.06-25.36)	<.001	
	Non-Hispanic Black: US vs TS	4.19 (2.03-8.65)	<.001	
	Non-Hispanic White: US vs TS	2.24 (1.59-3.15)	<.001	
	Other: US vs TS	12.22 (1.59-94.12)	.016	
Initiated	Race/ethnicity × rand group (interaction)	NA	.15	2.09 (1.28)
**Location**				
Identified	Location × rand group (interaction)	NA	.006	0.06 (0.04)
	Urban: US vs TS	5.47 (4.65-6.44)	<.001	
	Rural: US vs TS	13.60 (7.28-25.42)	<.001	
Confirmed	Location × rand group (interaction)	NA	.27	1.68 (0.75)
Initiated	Location × rand group (interaction)	NA	.35	1.61 (0.96)

Abbreviations: NA, not applicable; OR, odds ratio; rand, randomized; TS, targeted screening; US, universal screening.

^a^ Identified (major depressive disorder symptoms): positive Patient Health Questionnaire-9 (score >10 or response ≥1 on question 9) or behavior prompting referral to Student Assistance Program for major depressive disorder; confirmed: the Student Assistance Program does not diagnose, but to meet criteria, the Student Assistance Program must confirm identified behavior/symptoms warrant further evaluation; initiated: participated in at least 1 Student Assistance Program–recommended treatment/service for major depressive disorder, eg, follow-up mental health supports.

## Discussion

In a large, statewide RCT of schools with a diverse student body, universal school-based depression screening successfully doubled student initiation of MDD treatments and services, providing clear evidence of the benefit to addressing USPSTF screening beyond the clinic walls. These estimates are likely conservative owing to COVID-19–related school closures limiting screening completion (7% of students). Additionally, some positively screened students were directly referred to treatment, bypassing SAP, or were already in treatment, which precluded SAP referral. We conservatively counted these students as not meeting our primary outcome. For these reasons, the actual number of students successfully identified and treated for MDD is likely higher than recognized by our results.

Subgroup analysis by sex identified more positively screened female students, consistent with national data.^1^ However, finding no difference in treatment initiation by sex is inconsistent with national data.^30^ The previously noted data limitations may have influenced these findings. Subgroup analysis by race and ethnicity similarly found no differences in treatment initiation, different from national data,^30,31,32^ suggesting universal screening may increase treatment initiation for populations less likely to engage with behavioral health services. Non-Hispanic Black students had higher rates of identification by targeted screening that equalized with universal screening, suggesting that universal screening may reduce racial and ethnic bias in behavior-based referrals and support equity efforts in schools.^33^

In more than 40% of cases with SAP-recommended services, the primary reason for failure of treatment initiation was lack of parent or student consent. Parents may be less likely to consent to treatment unless student school functioning is jeopardized. This finding identifies a need to increase parent and adolescent mental health literacy, address stigma, and streamline parental consent processes. The results have significant policy implications for state legislation regarding adolescent mental health screenings, consent, and follow-up services.^34,35,36^

Prior studies of PHQ screening among Pittsburgh-area middle and high school students^9^ and in a southwestern US school-based clinic^10^ increased identification and referral. The Developmental Pathways Screening Program identified emotional difficulties at the middle school transition; 15% were positive.^12^ However, none of these studies assessed treatment initiation, a critical component of program effectiveness.

Columbia University’s Teen Screen identified mental health needs with screen-positive rates of 17%.^11,37^ Husky et al^13^ used Teen Screen's model in a mental health screening RCT of 656 students at 4 Pennsylvania schools with SAP referral for identified students. Similar to SHIELD’s findings, screened students were more likely to be referred to mental health services and access services, although disparities in treatment were not addressed.^13^

Our results differ from the RCT by Guo et al^14^ that found no effect of universal screening on treatment initiation. However, that study had a smaller sample size and younger students. Screenings were done with the PHQ-Adolescent version, which includes 2 additional items regarding suicidal thoughts.^9,14,38^ A positive result was defined as the top 20% of results for the school (vs using individual student scores).^14^ Schools also had a large percentage of Asian students, who are less likely to engage in mental health treatment.^14,30,39,40^

### Strengths and Limitations

To our knowledge, this was the first large-scale RCT to focus exclusively on the USPSTF recommendation for MDD screening among high school students within the school setting and to evaluate the effect on treatment initiation. Additional strengths include a diverse student population and use of existing school resources and team-based processes for referral and follow-up, supporting the reproducibility of this model.

The data are limited by what information schools provided. The team clarified missing or discrepant information, but outside referrals are not routinely logged by SAP, so this information was unavailable. The COVID-19 pandemic affected universal screening completion, which biased results toward the null. Study staff involvement may have improved the number of students successfully screened. Though trained, SAP staff are not mental health professionals and were not blinded to study group, so it is possible that biases affected the management of students identified in universal vs targeted screening. Participating schools represented a diverse group of students but were contacted based on prior relationships, responded to initial inquiries, and felt their SAP teams were well equipped to handle increased referrals. The effect of screening in schools with less confidence in their SAP teams may be different. However, this self-selection is consistent with USPSTF guidelines indicating screening should be done in the context of adequate capacity to ensure follow-up.^2^

## Conclusions

The results of this RCT support that universal adolescent MDD screening conducted in a school system can successfully identify students who would not otherwise be detected, with increased odds of treatment initiation among identified students. Schools with strong SAP teams or comparable infrastructure are arguably the appropriate target for this screening. Future work should identify the barriers to school participation in universal screening and the means to overcome them, including an economic and resource analysis in partnership with policy makers, schools, and parents to consider the most effective means for implementation nationally.

## Notes

Supplement 1.
Trial Protocol

Supplement 2. **eTable 1.** Definitions of Each Criterion That Comprise the Primary Outcome **eTable 2.** Reasons Students Did Not Meet Criterion 2 (Confirmed) Among Those Who Met Criterion 1 (Identified) **eTable 3.** Reasons Students Did Not Meet Criterion 3 (Initiated) Among Those Students Who Met Criterion 2 (Confirmed) **eTable 4.** Percentage of Students With Each Outcome by Subgroups of Sex, Race/Ethnicity, and School Location

Supplement 3.
Data Sharing Statement
