# Supplementary material for: Screening in High Schools to Identify, Evaluate, and Lower Depression Among Adolescents: A Randomized Clinical Trial
Source: JAMA Netw Open. 2021 Nov 5;4(11):e2131836. doi: 10.1001/jamanetworkopen.2021.31836 (PMC8571659; doi:10.1001/jamanetworkopen.2021.31836)
Supplement: Supplement 1. — Trial Protocol [file jamanetwopen-e2131836-s001.pdf]

This supplement contains the following items:
Original protocol including statistical analysis plan, final protocol, summary of changes.

HRP-591 - Protocol for Human Subject Research

Protocol Title:

Randomized Controlled Trial of Universal vs. Targeted School Screening for Adolescent Major Depressive Disorder

Principal Investigator:

Name: Deepa L. Sekhar
Department: Pediatrics
Telephone: 717-531-8006
E-mail Address: dsekhar@pennstatehealth.psu.edu

Version Date:

August 16, 2018

Clinicaltrials.gov Registration #:

This study will be registered as a clinical trial, as recommended by HSPO staff.

Important Instructions for Using This Protocol Template:

1. Add this completed protocol template to your study in CATS IRB (http://irb.psu.edu) on the "Basic Information" page, item 7.
2. This template is provided to help investigators prepare a protocol that includes the necessary information needed by the IRB to determine whether a study meets all applicable criteria for approval.
3. **Type your protocol responses below the gray instructional boxes of guidance language. If the section or item is not applicable, indicate not applicable.**
4. **For research being conducted at Penn State Hershey or by Penn State Hershey researchers only, delete the instructional boxes from the final version of the protocol prior to upload to CATS IRB (http://irb.psu.edu). For all other research, do not delete the instructional boxes from the final version of the protocol.**
5. When making revisions to this protocol as requested by the IRB, please follow the instructions outlined in the Study Submission Guide available in the Help Center in CATS IRB (http://irb.psu.edu) for using track changes.

If you need help…	
**University Park and other campuses:**<br>Office for Research Protections Human Research Protection Program<br>The 330 Building, Suite 205<br>University Park, PA 16802-7014<br>Phone: 814-865-1775<br>Fax: 814-863-8699<br>Email: irb-orp@psu.edu	**College of Medicine and Hershey Medical Center:**<br>Human Subjects Protection Office<br>90 Hope Drive, Mail Code A115, P.O. Box 855<br>Hershey, PA 17033<br>(Physical Office Location: Academic Support Building Room 1140)<br>Phone: 717-531-5687<br>Fax number: 717-531-3937<br>Email: irb-hspo@psu.edu

Table of Contents

1.0 Objectives

2.0 Background

3.0 Inclusion and Exclusion Criteria

4.0 Recruitment Methods

5.0 Consent Process and Documentation

6.0 HIPAA Research Authorization and/or Waiver or Alteration of Authorization

7.0 Study Design and Procedures

8.0 Subject Numbers and Statistical Plan

9.0 Confidentiality, Privacy and Data Management

10.0 Data and Safety Monitoring Plan

11.0 Risks

12.0 Potential Benefits to Subjects and Others

13.0 Sharing Results with Subjects

14.0 Subject Stipend (Compensation) and/or Travel Reimbursements

15.0 Economic Burden to Subjects

16.0 Resources Available

17.0 Other Approvals

18.0 Multi-Site Research

19.0 Adverse Event Reporting

20.0 Study Monitoring, Auditing and Inspecting

21.0 Future Undetermined Research: Data and Specimen Banking

22.0 References

1.0 Objectives

1.1 Study Objectives

OBJECTIVE1. Partnering with 8-12 PA public high schools (letters of commitment obtained) serving an estimated 9,650 predominantly minority, rural and/or low socioeconomic status (SES) students, we will conduct a randomized controlled trial, with student randomization by grade, to compare the effectiveness of universal school-based screening for adolescent major depressive disorder (MDD) versus the existing process of targeted screening based on concerning behavior.

- *Hypothesis 1: Universal screening will increase the number of adolescents with MDD screened, identified and engaged in treatment (MDD composite).*
- *Hypothesis 2 (Moderating): Universal screening will increase MDD screening, identification and treatment engagement for minorities.*
- *Hypothesis 3 (Moderating): Universal screening will increase the historically poorer rates of MDD screening, identification and treatment engagement for females*

OBJECTIVE 2. Analyze the impact of MDD screening on secondary outcomes currently collected by the school's Student Assistance Program (SAP) and the school district. These data will be obtained in aggregate by grade level at each participating school (a total of 32 data points for each item; 4 grades x 8 schools).

- *Hypothesis 1. Rates of missed schools days will improve in school populations with universal MDD screening secondary to earlier identification, treatment referral and engagement.*
- *Hypothesis 2. Grade point averages (GPA) will be improved among school populations with the universal MDD screening approach.*
- *Hypothesis 3. Rates of grade advancement/graduation will be improved among school populations with the universal MDD screening approach.*
- *Hypothesis 4. Student school policy violations and suspensions will remain unchanged among school populations with the universal MDD screening approach.*

1.2 Primary Study Endpoints

MDD composite which includes MDD screen positive for the universal screening arm (or concern in the targeted screening group),MDD identification, and MDD treatment engagement. Each of the primary study endpoints will be collected at the individual level by the school district by the end of the school year. No identifiable information will be collected by study staff.

1.3 Secondary Study Endpoints

The secondary endpoints listed below are individual level data points that will be obtained from the school district and/or SAP. No identifiable information will be collected by study staff.

MDD screen positive
MDD concern prompting Student Assistance Program triage
Suicidal adolescent (includes suicidal thoughts [positive response to PHQ-9 item 9], attempts and completed)
MDD identification
MDD treatment engagement

The secondary endpoints listed below are aggregate data points that will be obtained from the school district and/or SAP. No identifiable information will be collected by study staff.

Grade point averages
Attendance
Grade advancement or graduation rates
School policy violations and/or suspensions

Additional data points to be obtained for grades 9-12 include:

- At the individual level, the following moderator variables will be collected (each obtained from the school districts) :
    - Ethnicity - Hispanic vs. non-Hispanic
    - Race - white, black or other
    - Sex - male or female
- At the aggregate level, the following mediator variables will be collected:
    - District socioeconomic status - dichotomized into <50% vs. >50% based on data from PA School Performance Profile
    - Urban vs. rural – school districts will be categorized as urban vs. rural based on the definition applied by The Center for Rural PA, a Legislative Agency of the PA General Assembly
    - School size – school enrollment will be included as a continuous variable. Data will be obtained from school districts based on enrollment as of October 1 of the RCT
    - Ratio of guidance counselors/students – The ratio of counselors to students will be obtained from each school district
    - School-based mental health services – schools will be categorized by the availability of school-based mental health services (yes vs. no) based on information obtained from each school district.

We need to collect data at the individual level for 3 main reasons. First, we expect approximately 20% of students to opt out from the study, with opt out rates varying by grade. Aggregate data would necessarily include outcomes for students who are not enrolled in the study. This is particularly problematic for the universal screening group because students who opt out will not be offered the depression screening tool (PHQ-9). Second, we need to obtain gender and race/ethnicity to conduct important planned secondary analyses (subgroup analyses) that will examine efficacy of universal screening by these groups. In particular, we expect that females and minority students will have much higher rates of major depression disorder identified in the universal screening group. Third, in the universal screening group, we will be able to link responses to the PHQ-9 to outcomes, which will allow for estimation of important measures such as the false positive rate of the PHQ-9 (score ≥11, but SAP process determines no further referrals are needed). These measures will inform decision-making regarding the potential for implementation of the intervention (in other schools) should the results of the trial ultimately show efficacy.

2.0 Background

2.1 Scientific Background and Gaps

The prevalence of annual major depressive disorder (MDD) episodes among US adolescents rose from 8.3% in 2008 to 12.5% in 2015.[1] Close to 30% of adolescents with MDD reported suicidality in the prior year, with more than one in ten making a suicide attempt.[2] As a result, suicide was the 2nd leading cause of death among youth 10-24 years of age as of 2014.[3] Baseline data from HealthyPeople.gov found that only 2.1% of adolescent primary care office visits included depression screening in the years 2005-2007.[4] Additionally, Zenlea et al's work found primary care adolescent MDD screening was 80% less likely for Hispanic compared to non-Hispanic white adolescents.[5] Similar inequalities were reported for women who are three times more likely to have MDD, but less likely to be treated than males.[1,2,6] In response to the growing mental health crisis, the US Preventive Services Task Force (USPSTF) endorsed

universal screening for adolescent MDD in primary care in 2009.[7,8] The HealthyPeople.gov 2020 goal is a 10% increase in screening to a rate of 2.3%, which fails to address this adolescent public health crisis.[4] The USPSTF universal MDD screening recommendation was based on evidence that treatment of MDD is associated with moderate benefit.[7,8] While most experts in family medicine, pediatrics, psychology and child psychiatry agree that surveillance of adolescents at high-risk for MDD is warranted, the USPSTF updated their recommendations in 2016 with a call to address several knowledge gaps:

1) Does screening increase the proportion of adolescents identified with MDD?
2) What are the benefits and unintended consequences of MDD screening for subgroups: age, sex, race, ethnicity and socioeconomic status (SES)?
3) What are the benefits and unintended consequences of screening in nonclinical settings?[8]

We propose that schools may provide an effective setting to conduct universal MDD screening. While over half of US adolescents do not have annual preventive health visits, regardless of race, ethnicity or SES most attend school.[9] The regular contact with schools compared to contact with the medical setting has been used to advocate for many school-based universal health screenings that impact academic success (e.g. vision, hearing). However, while current school screenings address multiple physical health domains, none address mental health.[10] Targeted mental health screening is the current school process for students who display signs concerning for MDD and results in referral to the school's Student Assistance Program (SAP). SAP operates in all 500 Pennsylvania school districts and functions similar to a triage service by assessing symptom severity, and then if appropriate, providing referrals to school or community-based mental health resources.[11] Students may self-refer, but all other SAP referrals depend upon a student exhibiting concerning behavior that is detected by school staff, peers or parents, which results in a targeted screening process with obvious limitations.

2.2 Previous Data

Our research team is acutely aware of the concerns the topic of adolescent depression screening may raise among school staff, providers, parents and adolescents. We have had ample opportunity in our pilot work to discuss and address many of these issues as outlined below. First, from April-Sept 2016 we conducted eight focus groups (7-10 participants each, n=62) to better understand the perspective of key stakeholders regarding Whole Child Health, specifically the importance of both physical and mental wellness. These focus groups included 2 parent groups, 2 school nurse groups, 2 groups of school teachers and administrators and 2 groups of medical providers (pediatrics and family medicine). The work was funded by the Penn State Social Science Research Institute-Children, Youth and Families Consortium. Participant conversations were instrumental in shaping the current proposal. In addition, following the aforementioned focus groups, we conducted a Community Engagement Studio in August 2017 funded by the Penn State Center for Translational Science Institute. These 2 hour sessions are specifically intended to inform grant proposals that depend upon successful community engagement. Participant perspectives ranged from adolescent to parent, school staff, the leaders of two mental health and suicide prevention organizations (Aevidum and the Jana Marie Foundation), a Behavioral Health Managed Care company representative and the project director of Pennsylvania's Garrett Lee Smith Youth Suicide Prevention Grant in addition to our Penn State Research team.

2.3 Study Rationale

Rates of major depression are rising among US adolescents paralleled by a rise in the rate of adolescent suicides. The most recent data indicates that 1 in 8 adolescents (12.5%) experienced an MDD episode in the past 12 months.[1] From 2005-2014 a significant increase in MDD episode trends was observed for all racial and ethnic groups except Non-Hispanic Blacks who demonstrated a smaller, statistically nonsignificant increasing trend.[6] The most striking increase in MDD trends was for females across all racial and ethnic groups. Adolescent females demonstrated rates of MDD episodes over 3 times that of males (19.5% vs. 5.8%).[1,6] Some authors have suggested that adolescent females have increased exposure to depression risk factors including cyberbullying, mobile phone use and texting.[6] Along with

the rise in MDD episodes, adolescent females have demonstrated a significant rise in emergency department visits for nonfatal self-inflicted injuries with rates since 2009 increasing by 19% annually from 110 in 2009 to 318 per 100,000 in 2015.[12] Self-inflicted injury is one of the strongest risk factors for suicide, and the suicide rate for female adolescents reached its highest in the past 40 years according to 2017 Centers for Disease Control and Prevention data.[13,14]

Adolescent MDD has negative effects on academic performance, with increasing severity of depressive symptoms linked to a lower grade point average as well as subjective assessments of increased school workload and concentration difficulties. Adolescents with untreated MDD experience poorer interpersonal relationships, lower self-esteem, social isolation, and increased risk-taking behaviors including substance use, as well as multiple physical and mental health comorbidities in adulthood.[7,8,15-17] For 60-90% of adolescents, symptoms of a MDD episode may remit within in a year. The larger problem is that 50-75% of these adolescents will develop subsequent MDD episodes within 5 years, resulting a chronic or relapsing disorder.[18] Studies also suggest that recovery is not complete between episodes, with most individuals reporting residual symptoms or impairment.[18]

Despite the rising rates of depression, there has been no change in mental health treatment among adolescents with a MDD episode from 2005-2014.[6] Only 36-44% of children and adolescents with MDD receive treatment, underscoring that MDD is underdiagnosed and undertreated.[6] This disparity is especially pronounced for disadvantaged populations. Specifically, Black and Hispanic adolescents are significantly less likely to be diagnosed with mental illness and referred for treatment compared to their White counterparts.[5,19] These disparities often stem from lack of access to primary care.[20] Even for those who have a primary care provider, data from HealthyPeople.gov indicates a steady decline in rates of screening with only 1.4% of primary care office visits including MDD screening as of 2009-2011.[4] In addition to minorities, rural youth and those of lower SES receive fewer preventive care services than their white, urban, high SES counterparts, further limiting their access to MDD screening in the context of well-care.[20,21]

3.0 Inclusion and Exclusion Criteria

3.1 Inclusion Criteria

Students in grades 9-12 at eight public schools [redacted] in Pennsylvania that previously committed to partner with us in this project. HRP-504- School Permission to Conduct Research forms will be included in our CATS application documentation for each participating district to show district approval of the opt out procedure and to agree with their 3rd Party Protection of Pupil Rights Amendment (PPRA) policies. Further, included as supporting documents are letters from [redacted] who have all acknowledged and shown support of the opt out approach.

3.2 Exclusion Criteria

Students whose parents complete the opt-out consent
Students not enrolled in one of the participating 8 schools
Students not in grades 9-12
Students with disabilities that are deemed unable to participate by the school district

3.3 Early Withdrawal of Subjects

3.3.1 Criteria for removal from study

N/A

3.3.2 Follow-up for withdrawn subjects

N/A

4.0 Recruitment Methods

4.1 Identification of subjects

Eight PA public schools committed to partner with us to complete the proposed work. PI [redacted] and co-investigator [redacted] have previously partnered with several of these school districts through their prior research. In total, the schools serve over 9,000 students and were selected as they represent a large number of minorities, low SES and/or rural student populations who have known disparities in mental health services.

4.2 Recruitment process

After discussion with our stakeholders and the Penn State Institutional Review Board, we will pursue opt-out consent for screening given the importance of MDD screening and the low-risk aside from identification of a suicidal student. Parents will be informed of their child's enrollment in screening and given the opportunity to opt out prior to the fall intervention. For this proposal, in cases of a shared custody agreement, if either parent or guardian opts out of the study, the student will be considered ineligible for enrollment. In this case, no information will be collected, even in the case of students randomized to the targeted screening arm, which is the usual school process. Also, any participating student randomized to the universal screening arm who does not assent at the time of screening will not be required to complete the screening form. Students 18 years and older are anticipated to be a small minority of the students at the start of the academic year, but they will also have the opportunity to opt out of study involvement if desired.

4.3 Recruitment materials

No recruitment materials will be needed for this study. However, a letter will be sent home to parents to inform them of their child's enrollment in the screening and given the opportunity to opt out prior to the intervention. If they do not wish to participate, parents will be asked to return the Opt-Out form in-person or by mail. Opt-out letters will be printed on district letterhead to include the participating school's mailing address. Parents may also return the form signed and scanned, if the school decides to send the letter via email. A copy of the PHQ-9 questionnaire will be included in the letter sent to the parents. Additionally, the opt-out letter will be translated into a Spanish by the Department of Care Coordination and provided to each school.

4.4 Eligibility/screening of subjects

N/A

5.0 Consent Process and Documentation

5.1 Consent Process

5.1.1 Obtaining Informed Consent

5.1.1.1 Timing and Location of Consent

N/A

5.1.1.2 Coercion or Undue Influence during Consent

N/A

5.1.2 Waiver or alteration of the informed consent requirement

We are requesting a waiver of the informed consent requirement. We are pursuing opt-out consent for screening given the important of MDD screening and the low-risk aside from identification of a suicidal student. At 14 years of age, PA youth are eligible to consent to mental

health services without parental consent. Our use of the opt out is really intended to include and engage the parents and communities we are working with. The opt out will be a letter sent home to parents (either via email or regular mail as per school preference) giving them the option to decline participation for their student. Schools will be responsible for tracking students whose parents have opted-out. In addition, students in the universal arm will have the option to decline participation themselves on the screening day via the iPad handed to them. The first screen will describe PHQ-9; inform students that participation is voluntary; and participation may be stopped at any time and will not affect their school standing or grades.

5.2 Consent Documentation

5.2.1 Written Documentation of Consent

N/A

5.2.2 Waiver of Documentation of Consent (Implied consent, Verbal consent, etc.)

N/A

5.3 Consent – Other Considerations

5.3.1 Non-English Speaking Subjects

A Spanish translator was included in the grant and will join the research team for the MDD screening at schools where these services would be needed. In addition, the opt out information will be translated and sent in Spanish for those parents who the school indicates would have trouble with the English version forms. The assent will also be translated into Spanish for those students who indicate that Spanish is their preferred language. The PHQ-9 is already available in multiple languages including Spanish.

5.3.2 Cognitively Impaired Adults

N/A

5.3.2.1 Capability of Providing Consent

N/A

5.3.2.2 Adults Unable To Consent

N/A

5.3.2.3 Assent of Adults Unable to Consent

N/A

5.3.3 Subjects who are not yet adults (infants, children, teenagers)

5.3.3.1 Parental Permission

We are requesting a waiver of informed consent. Rather, parents/guardians will receive an opt-out form. We are pursuing opt-out consent for screening given the important of MDD screening and the low-risk aside from identification of a suicidal student. At 14 years of age, PA youth are eligible to consent to mental health services without parental consent. Our use of the opt out is really intended to include and engage the parents and communities we are working with.

5.3.3.2 Assent of subjects who are not yet adults

Students in the universal arm will have the option to decline participation themselves on the screening day via the iPad handed to them. The first screen will describe PHQ-9; inform students that participation is voluntary; and participation may be stopped at any time and will not affect their school standing. The completion of the PHQ-9 implies a student's voluntary consent to participate in the research. Students who decide not to participate will not complete the PHQ-9, but will still be tracked similar to students randomized to the targeted screening arm. The study team will obtain their demographic information and the student will be followed through the academic year for SAP triage intakes initiated by the standard pathway (concern by teachers, nurse, parent, peer, or self-referral), any referrals and treatment engagement. No identifiable information will be obtained and it will be noted in study records that the student did not assent to participate in the MDD screener. A copy of this assent form is included in the consent form section.

6.0 HIPAA Research Authorization and/or Waiver or Alteration of Authorization

6.1 Authorization and/or Waiver or Alteration of Authorization for the Uses and Disclosures of PHI

Check all that apply:

☒ **Not applicable, no identifiable protected health information (PHI) is accessed, used or disclosed in this study.** *[Mark all parts of sections 6.2 and 6.3 as not applicable]*

☐ **Authorization will be obtained and documented as part of the consent process.** *[If this is the only box checked, mark sections 6.2 and 6.3 as not applicable]*

☐ **Partial waiver is requested for recruitment purposes only (Check this box if patients' medical records will be accessed to determine eligibility before consent/authorization has been obtained).** *[Complete all parts of sections 6.2 and 6.3]*

☐ **Full waiver is requested for entire research study (e.g., medical record review studies).** *[Complete all parts of sections 6.2 and 6.3]*

☐ **Alteration is requested to waive requirement for written documentation of authorization (verbal authorization will be obtained).** *[Complete all parts of sections 6.2 and 6.3]*

6.2 Waiver or Alteration of Authorization for the Uses and Disclosures of PHI

6.2.1 Access, use or disclosure of PHI representing no more than a minimal risk to the privacy of the individual

6.2.1.1 Plan to protect PHI from improper use or disclosure

N/A

6.2.1.2 Plan to destroy identifiers or a justification for retaining identifiers

N/A

6.2.2 Explanation for why the research could not practicably be conducted without access to and use of PHI

N/A

6.2.3 Explanation for why the research could not practicably be conducted without the waiver or alteration of authorization

N/A

6.3 Waiver or alteration of authorization statements of agreement

N/A

7.0 Study Design and Procedures

7.1 Study Design

Due to the timeline for this funding opportunity, with an anticipated July 2018 start, 4 schools will engage in the randomized control trial (RCT) in study year 1 and the additional 4 schools in study year 2. Many large scale randomized control trials do not enroll all participants at one time point, and in many cases enrollment occurs slowly over the course of several years. We do not anticipate there will be major changes over the course of two academic years that would significantly alter the results compared to conducting the RCT in the same academic year, especially because students will be randomized within schools. Finally, staggering enrollment will give the research team the opportunity to troubleshoot any unanticipated issues with the first 4 participating schools.

7.2 Study Procedures

7.2.1 Enrollment

Parents will be informed of their child's enrollment in screening and given the opportunity to opt out prior to the fall intervention. The opt out will be a letter sent home to parents (either via email or regular mail as per school preference) giving them the option to decline participation for their student. The study team will provide each school with unique study IDs to be assigned to every student, grades 9-12. Study IDs will include 8 numbers, the first two representing the school, the second two the grade and the remaining 4 will be unique to each participating student. Schools will be required to assign these unique study IDs to each student, grades 9-12. Schools will complete a linking list (spreadsheet template) ensuring that all students, grades 9-12 are included. A completed linking list will include student names (first and last), PASECURED ID, unique study ID and demographics information (grade, age, sex, race and ethnicity). Schools will remove student names and PASECUREID before sending the spreadsheet to the study team. *The full linking list (including student name and PASECURIEDID) will remain on the school's spreadsheet and in the school's possession for tracking of study outcomes through the year.* By using a unique study ID for each student, the study team will never receive identifiable information of students. If a parent returns the opt-out form, school staff will still assign a student a unique study ID but no demographic information will be included. This is so the study team may properly report the number of opt-out letters returned. This procedure ensures that students whose parents have opted-out are not tracked throughout the year.

At the time of the actual universal screening students will also be provided the chance to opt out by clicking the appropriate opt out box on the iPad handed to them. We anticipate this will lead to study enrollment of 80% of eligible students. The first screen will describe PHQ-9 and that proceeding is voluntary. The completion of the PHQ-9 implies a student's voluntary consent to participate in the research. Unless the parent opt out is returned, participants in both arms will be followed through the school year for SAP triage, follow-up referrals and treatment engagement. Those students in the universal screening arm with PHQ-9 scores $\geq$ 11 (MDD screen positive) corresponding to moderate depressive symptoms, will proceed through the standard process for anyone referred by traditional means to a SAP triage interview. The student will either be referred to appropriate community or

school-based treatment or SAP will determine no follow-up is needed. For those who are recommended to additional services by SAP, treatment engagement will be tracked per current SAP processes. The study team will receive individual level outcome data from the school district, containing no identifiable information.

7.2.2 Randomization

We will randomize by grade levels within each school to receive either one-time universal screening (via PHQ-9) or targeted screening (current SAP process). For the 8 schools included in the study, 4 schools (50%) will be randomized such that students in 9th and 11th grades will receive universal screening and students in 10th and 12th grades will receive targeted screening, and 4 schools (50%) will be randomized such that students in 9th and 11th grades will receive targeted screening and students in 10th and 12th grades will receive universal screening. Randomization will be done only for students whose parents do not signal an unwillingness to participate in the study ("opt out"). Students and study personnel will not be blinded to randomized group at each school site. Randomization will be done by grade level primarily for pragmatic reasons because many PA health-based screenings are grade-specific (e.g. hearing screen in 11th grade) and screenings for a grade occur at the same time. The study will be conducted within schools, but it is not a cluster randomized study, in which an entire school (cluster) is assigned to one of the study groups. A cluster randomized study was considered but ultimately not pursued because randomization within schools controls for (1) within-school (community) factors that may contribute to higher or lower rates of SAP referral, (2) differences in school sizes, and (3) potential differences in rates of parental opt out among schools. These benefits were balanced against the concern of potential contamination between study groups, whereby those in the targeted screening group may benefit from the school-wide push to conduct universal screening.

7.2.3 Universal MDD Screening Arm (Intervention – Treatment)

Students randomized to the universal screening arm will complete a PHQ-9. This screening tool includes nine close-ended questions with a scoring system ranging from 0 to 27. The PHQ-9 screens will be administered on an iPad with an internet connection which allows direct entry of the results in REDCap. To prevent the duplication of unique study IDs, the study team will have entered all unique IDs into REDCap prior to the screening. A list of participant IDs will be provided to the school member present during the screening. Once the corresponding unique study ID has been entered, the study staff will give the iPad to the student to assent and complete the PHQ-9. This process will ensure that the correct study ID is used and prevent duplication of study IDs. Further, this will safeguard against any student from participating whose parent returned the opt-out letter. No names or other identifying information will be used. Paper copies of the assent and PHQ-9 will be available as a backup should problems arise with internet connectivity. The same measures will be taken to ensure no names or other identifying information is used or collected.

In order to immediately identify suicidal intent the survey will be set to flag positive questions in real time. When students have completed the PHQ-9 screen the REDCap survey will prompt them to hand the completed questionnaire to study staff, who will see a screen indicating a positive flag. Students with suicidal intent (question #9 any response besides "Not at all") will receive immediate evaluation and referral to emergency care as per current school protocols. PA schools are required to have a plan to address suicidal students (Act 71). A suicidal participant identified during the screening would not be allowed to leave the screening area unless accompanied by appropriate school or research staff. This student would then proceed through the standard school pathway for managing a student with intentions of self-harm. To ensure school staff is comfortable to manage a student in crisis and that this persists beyond the period of the grant, at least 5 staff per school in addition to at least 4 Penn State research staff will complete online evidence-based suicide prevention training (Question, Persuade, and Refer [QPR] Suicide Triage Training). In addition, all school crisis plans will be carefully reviewed with staff following the training to ensure the steps are realistic and staff is comfortable to execute the plan.

The district identified, QPR trained staff member will be available at the time of screening. Study staff who are also QPR trained, will be present during the screening.

7.2.4 Targeted Screening Arm (Current Process – Control)

Students randomized to the targeted screening arm will complete their routine school-based screenings. Students will be followed through the academic year for SAP triage intakes initiated by the standard pathway (concern by teachers, nurse, parent, peer, or self-referral), any referrals and treatment engagement.

7.2.5 Sharing Screening Results

At 14 years of age, PA youth are eligible to consent to mental health services without parental consent, therefore, screening results will not be shared with parents. Those students with scores $\geq$ 11 (MDD screen positive) corresponding to moderate depressive symptoms will, however, proceed through the standard process for anyone referred by traditional means to a SAP triage interview. The student will either refer to appropriate community or school-based treatment or therapy (MDD identified) or SAP will determine no follow-up is needed. For those who are recommended to additional services by SAP, treatment engagement will be tracked per current SAP process. As per current school policy, students with suicidal feelings will receive immediate referral to emergency care and parents will be notified by the school.

7.3 Duration of Participation

The student's participation is limited to the 5 minutes it takes for them to participate in the screening process.

8.0 Subject Numbers and Statistical Plan

8.1 Number of Subjects

The 8 schools to be included in the study have an estimated total enrollment of approximately 9650 students. The overall rate of parental opt out is expected to be around 20%, resulting in approximately 7720 students included in the study. We assumed a 15% attrition rate for students who move, drop out of school, or opt out later in the school year. Thus, the study is expected to include 6550 students.

8.2 Sample size determination

A total of 3275 students in each randomized group (an overall sample size of 6550) yields >99% statistical power to detect a difference of 3% versus 6% using a 2-sided test conducted at a Type I error rate of 5% in a mixed effect logistic regression model.

8.3 Statistical methods

The principles of intention-to-treat (ITT) will be used for all statistical analyses related to primary and secondary aims. For the primary aim comparing universal to targeted screening, the statistical analysis will be conducted using a mixed effects logistic regression model. The primary outcome, MDD composite, will be an indicator whether a student was screen positive (or concerns raised in the targeted screening arm), identified as having MDD and subsequently engaged in treatment (1=yes, 0=no). The model will include a fixed effect for randomized group (0=targeted screening, 1=universal screening) and a random effect for school. The random effect accounts for correlation among students enrolled within the same school. The primary parameter of interest will be the log odds of MDD composite in the universal screening group compared to the targeted screening group. Statistical significance of the log odds will be assessed using a 2-sided Wald test. Point estimates for the odds ratio along with a 95% confidence interval will be reported.

For the analysis of Hypotheses 2 and 3 (Objective 1) evaluating universal screening and targeted screening by selected subgroups, the same mixed effects logistic regression modeling framework will be used, but the model will be extended by including a fixed effect for subgroup and an interaction effect for subgroup by randomized group. The interaction term will be the parameter of interest. A significant interaction term indicates that rates of MDD treatment engagement for universal versus targeted screening differ by subgroup level (e.g. female vs. male). For the analysis of Hypotheses 1 through 4 (Objective 2) that evaluates universal screening and targeted screening based on school SAP data, we will have only 32 total data points (4 grades in each of 8 schools). Mixed-effects linear (continuous outcomes) and logistic regression (binary outcomes) will be used, as appropriate, with a fixed effect for randomized group and a random effect for school. The parameter of interest will be the log odds for the universal compared to the targeted screening group. Due to the smaller sample size used for these outcomes, these analyses will be considered to be primarily hypothesis-generating. Potential mediating variables (socioeconomic status, rural vs. urban location, school size, ratio of guidance counselors to students and availability of school-based mental health services) will be evaluated for both the Objective 1 and 2 hypotheses.

Additional secondary outcomes will include MDD screen results, MDD concern prompting Student Assistance Program triage, MDD identification and MDD treatment engagement analyzed individually (rather than as part of MDD composite). Finally any suicidal adolescents (suicidal thoughts [positive response to PHQ-9 item 9], attempts and completed) will be analyzed as a secondary outcome.

We need to collect data for individuals for 3 main reasons. First, we expect approximately 20% of students to opt out from the study, with opt out rates varying by grade. Aggregate data would necessarily include outcomes for students who are not enrolled in the study. This is particularly problematic for the universal screening group because students who opt out will not be offered the depression screening tool (PHQ-9). Second, we need to obtain gender and race/ethnicity to conduct important planned secondary analyses (subgroup analyses) that will examine efficacy of universal screening by these groups. In particular, we expect that females and minority students will have much higher rates of major depression disorder identified in the universal screening group. Third, in the universal screening group, we will be able to link responses to the PHQ-9 to outcomes, which will allow for estimation of important measures such as the false positive rate of the PHQ-9 (score ≥11, but SAP process determines no further referrals are needed). These measures will inform decision-making regarding the potential for implementation of the intervention (in other schools) should the results of the trial ultimately show efficacy.

Efforts will be made to ensure completeness of data where possible, but missing data will occur for a number of anticipated reasons. First, a student may move during the course of the school year to another school district or drop out from school entirely. No data will be collected after this time point. Second, parents may opt out their child from the study at any time during the school year. Third, students who turn 18 during the school may decide to opt out of the study themselves. In both of these instances, data from SAP referrals that occur after opting out will not be collected for purposes of the study. Fourth, during the one-time universal screening phase, students may decide to leave data forms incomplete, including the PHQ-9. To decrease these instances, the PHQ-9 will be taken on an iPad program and the survey will alert if the form is left incomplete.

9.0 Confidentiality, Privacy and Data Management

Please see HRP-598 - Research Data Plan Review Form

10.0 Data and Safety Monitoring Plan

N/A: This study does not involve more than minimal risk to subjects, and the magnitude of harm/discomfort is not greater than that ordinarily encountered in daily life.

11.0 Risks

Risk involved in participating in this study are low aside from identification of a suicidal student, in which case measures are already currently in place in each school building to address.

12.0 Potential Benefits to Subjects and Others

12.1 Potential Benefits to Subjects

Potential benefit to subjects of positive screenings include a referral to SAP to receive support in managing their MDD.

12.2 Potential Benefits to Others

The potential public health impact of the proposed project cannot be overstated. MDD is a prevalent, disabling and a growing US public health problem. The problem is identified by national organizations focused on our country's health care priorities (Healthy People 2020, US Department of Health and Human Services). MDD leads both to functional impairment and higher rates of morbidity and mortality. In addition, MDD leads to significant social and economic consequences, including increased use of health resources and lost work productivity. A public health goal should include identification of those at risk for depression with the delivery of interventions to these individuals. Schools are a point of intervention with a high potential for early identification and prevention. Currently, fewer than 2 out of every 100 adolescents receives guideline-concordant major depression screening. We propose reaching nearly 80 of every 100 adolescents in the school setting, vastly increasing the identification of adolescents suffering from MDD with a goal to decrease both morbidity and mortality. Schools are an ideal partner for this approach, given their tremendous reach across the nation to nearly all adolescents.

13.0 Sharing Results with Subjects

Individual results will not be shared with other participants.

14.0 Subject Stipend (Compensation) and/or Travel Reimbursements

N/A

15.0 Economic Burden to Subjects

15.1 Costs

There are no financial costs associated with participating in this research.

15.2 Compensation for research-related injury

N/A

16.0 Resources Available

16.1 Facilities and locations

Screening will take place in each of the 8 school buildings previously identified.

16.2 Feasibility of recruiting the required number of subjects

All 8 school districts have already expressed interest in participating as evidenced through letters of support obtained during the proposal process. Current relationships through past and present programming with the school districts created feasibility for recruitment.

16.3 PI Time devoted to conducting the research

[redacted] will monitor the progress of the study during all phases and hold bi-weekly meetings with research staff.

16.4 Availability of medical or psychological resources

It is not anticipated that medical or psychological resources will be needed on site, given that study procedures are minimal risk. However, students with suicidal intent will receive immediate evaluation and referral to emergency care as per current school protocols. PA schools are required to have a plan to address suicidal students. A QPR trained staff member will be available at the time of screening. Students with a PHQ-9 score $\geq$11 will proceed to Student Assistance Program triage as per the standard of care by which students exhibiting concerning behavior (outbursts, declining grades) would be referred for assessment.

16.5 Process for informing Study Team

The investigators and project coordinator/study staff have completed their required Collaborative IRB Training Initiative (CITI) in the protection of human research subjects. The study team will be educated on the importance of confidentiality, and proper data handling and storage. Four study team members will also complete the Question, Persuade, Refer suicide triage training in order to assist the school staff as needed during the time of actual screening.

17.0 Other Approvals

17.1 Other Approvals from External Entities

N/A

17.2 Internal PSU Committee Approvals

Check all that apply:

- ☐ Anatomic Pathology – Hershey only – Research involves the collection of tissues or use of pathologic specimens. Upload a copy of HRP-902 - Human Tissue For Research Form on the "Supporting Documents" page in CATS IRB. This form is available in the CATS IRB Library.

- ☐ Animal Care and Use – All campuses – Human research involves animals and humans or the use of human tissues in animals

- ☐ Biosafety – All campuses – Research involves biohazardous materials (human biological specimens in a PSU research lab, biological toxins, carcinogens, infectious agents, recombinant viruses or DNA or gene therapy).

☐ Clinical Laboratories – Hershey only – Collection, processing and/or storage of extra tubes of body fluid specimens for research purposes by the Clinical Laboratories; and/or use of body fluids that had been collected for clinical purposes, but are no longer needed for clinical use. Upload a copy of HRP-901 - Human Body Fluids for Research Form on the "Supporting Documents" page in CATS IRB. This form is available in the CATS IRB Library.

☐ Clinical Research Center (CRC) Advisory Committee – All campuses – Research involves the use of CRC services in any way.

☐ Conflict of Interest Review – All campuses – Research has one or more of study team members indicated as having a financial interest.

☐ Radiation Safety – Hershey only – Research involves research-related radiation procedures. All research involving radiation procedures (standard of care and/or research-related) must upload a copy of HRP-903 - Radiation Review Form on the "Supporting Documents" page in CATS IRB. This form is available in the CATS IRB Library.

☐ IND/IDE Audit – All campuses – Research in which the PSU researcher holds the IND or IDE or intends to hold the IND or IDE.

☒ Scientific Review – Hershey only – All investigator-written research studies requiring review by the convened IRB must provide documentation of scientific review with the IRB submission. The scientific review requirement may be fulfilled by one of the following: (1) external peer-review process; (2) department/institute scientific review committee; or (3) scientific review by the Clinical Research Center Advisory committee. NOTE: Review by the Penn State Hershey Cancer Institute Scientific Review Committee is required if the study involves cancer prevention studies or cancer patients, records and/or tissues. For more information about this requirement see the IRB website at: http://www.pennstatehershey.org/web/irb/home/resources/investigator

18.0 Multi-Site Research

N/A

19.0 Adverse Event Reporting

19.1 Reporting Adverse Reactions and Unanticipated Problems to the Responsible IRB

In accordance with applicable policies of The Pennsylvania State University Institutional Review Board (IRB), the investigator will report, to the IRB, any observed or reported harm (adverse event) experienced by a subject or other individual, which in the opinion of the investigator is determined to be (1) unexpected; and (2) probably related to the research procedures. Harms (adverse events) will be submitted to the IRB in accordance with the IRB policies and procedures.

20.0 Study Monitoring, Auditing and Inspecting

20.1 Auditing and Inspecting

The investigator will permit study-related monitoring, audits, and inspections by the Penn State quality assurance program office(s), IRB, the sponsor, and government regulatory bodies, of all study related

documents (e.g., source documents, regulatory documents, data collection instruments, study data etc.). The investigator will ensure the capability for inspections of applicable study-related facilities (e.g., pharmacy, diagnostic laboratory, etc.).

21.0 Future Undetermined Research: Data and Specimen Banking

N/A

21.1 Data and/or specimens being stored

N/A

21.2 Location of storage

N/A

21.3 Duration of storage

N/A

21.4 Access to data and/or specimens

N/A

21.5 Procedures to release data or specimens

N/A

21.6 Process for returning results

N/A

22.0 References

1. Office of Disease Prevention and Health Promotion. Healthy People.gov. Mental Health and Mental Disorders. MNMD-4.1 Reduce the proportion of adoelscents aged 12 to 17 years who experience major depressive episodes. https://www.healthypeople.gov/2020/data-search/Search-the-Data#objid=4813; Updated December 11, 2017. Accessed December 11, 2017.
2. Avenevoli S, Swendsen J, He JP, Burstein M, Merikangas KR. Major depression in the national comorbidity survey-adolescent supplement: prevalence, correlates, and treatment. *J Am Acad Child Adolesc Psychiatry.* 2015;54(1):37-44.e2.
3. Heron M. Deaths: Leading causes for 2014. National Vital Statistics Reports. 2016;65(5). Hyattsville, MD: National Center for Health Statistics. https://www.cdc.gov/nchs/data/nvsr/nvsr65/nvsr65_05.pdf. Accessed January 3, 2018.
4. Office of Disease Prevention and Health Promotion. Healthy People.gov. Mental Health and Mental Disorders. MHMD-11.2. Increase the proportion of primary care physician office visits where youth aged 12 to 18 years are screened for depression. https://www.healthypeople.gov/2020/data-search/Search-the-Data#objid=4808; Updated December 11, 2017. Accessed December 11, 2017.
5. Zenlea IS, Milliren CE, Mednick L, Rhodes ET. Depression screening in adolescents in the United States: a national study of ambulatory office-based practice. *Acad Pediatr.* 2014;14(2):186-191.
6. Mojtabai R, Olfson M, Han B. National trends in the prevalence and treatment of depression in adolescents and young adults. *Pediatrics.* 2016;138(6):e20161878.
7. Forman-Hoffman V, McClure E, McKeeman J, et al. Screening for major depressive

disorder in children and adolescents: A systematic review for the U.S. Preventive Services Task Force. *Ann Intern Med.* 2016;164(5):342-349.
8. Siu AL, US Preventive Services Task Force. Screening for depression in children and adolescents: U.S. Preventive Services Task Force recommendation statement. *Ann Internal Med.* 2016;164(5):360-366.
9. Adams SH, Park MJ, Twietmeyer L, Brindis CD, Irwin CE, Jr. Association Between Adolescent Preventive Care and the Role of the Affordable Care Act. *JAMA Pediatr.* 2017 Nov 6. doi: 10.1001/jamapediatrics.2017.3140.
10. Pennsylvania House of Representatives. House Co-Sponsorship Memoranda. House of Representatives Session of 2015-2016 Regular Session. http://www.legis.state.pa.us/cfdocs/Legis/CSM/showMemoPublic.cfm?chamber=H&SPick=20150&cosponId=20186. Published May 6, 2016. Accessed January 3, 2018.
11. Student Assistance Program FY 2015-2016. Mental Health-Drug and Alcohol Statistical Data. https://sapjqrs.org/uploadedfiles/JQRS%20Exec%20Summary%202015-16.pdf. Accessed December 11, 2017.
12. Mercado MC, Holland K, Leemis RW, Stone DM, Wang J. Trends in Emergency Department Visits for Nonfatal Self-inflicted Injuries Among Youth Aged 10 to 24 Years in the United States, 2001-2015. *JAMA.*2017;318(19):1931-1933.
13. Centers for Disease Control and Prevention. QuickStats: Suicide Rates for Teens Aged 15-19 Years, by Sex – United States , 1975-2015. *MMWR Wkly Rep* 2017; 66(30):816. https://www.cdc.gov/mmwr/volumes/66/wr/mm6630a6.htm. Accessed December 11, 2017.
14. National Alliance on Mental Illness. Depression. http://www.nami.org/Learn-More/Mental-Health-Conditions/Depression. Accessed January 3, 2018.
15. Khoddam R, Jackson NJ, Leventhal AM. Internalizing symptoms and conduct problems: Redundant, incremental, or interactive risk factors for adolescent substance use during the first year of high school? *Drug Alcohol Depend.* 2016;169:48-55.
16. Fergusson DM, Woodward LJ. Mental health, educational, and social role outcomes of adolescents with depression. *Arch Gen Psychiat.* 2002;59(3):225-231.
17. Thapar A, Collishaw S, Pine DS, Thapar AK. Depression in adolescence. *Lancet.* 2012;379(9820):1056-1067.
18. Thomas JF, Temple JR, Perez N, Rupp R. Ethnic and gender disparities in needed adolescent mental health care. *J Health Care Poor Underserved.* 2011;22(1):101-110.
19. Lau M, Hua L, Flores G. Racial/ethnic disparities in health and health care among US adolescents. *Health Serv Res*. 2012;47(5):2031-2059.
20. Irwin CE, Jr., Adams SH, Park MJ, Newacheck PW. Preventive care for adolescents: few get visits and fewer get services. *Pediatrics.* 2009;123(4):e565-572.
21. Dixon SK, Hoopes AJ, Benkeser D, Grigg A, Grow HM. Characterizing key components of a medical home among rural adolescents. *Journal Adolesc Health.* 2016;58(2):141-147.

HRP-591 - Protocol for Human Subject Research

Protocol Title:
Randomized Controlled Trial of Universal vs. Targeted School Screening for Adolescent Major Depressive Disorder

Principal Investigator:
Name: Deepa L. Sekhar
Department: Pediatrics
Telephone: 717-531-8006
E-mail Address: dsekhar@pennstatehealth.psu.edu

Version Date:
May 15, 2020

Clinicaltrials.gov Registration #:
NCT03716869

Important Instructions for Using This Protocol Template:

1. Add this completed protocol template to your study in CATS IRB (http://irb.psu.edu) on the "Basic Information" page, item 7.
2. This template is provided to help investigators prepare a protocol that includes the necessary information needed by the IRB to determine whether a study meets all applicable criteria for approval.
3. **Type your protocol responses below the gray instructional boxes of guidance language. If the section or item is not applicable, indicate not applicable.**
4. **For research being conducted at Penn State Hershey or by Penn State Hershey researchers only, delete the instructional boxes from the final version of the protocol prior to upload to CATS IRB (http://irb.psu.edu). For all other research, do not delete the instructional boxes from the final version of the protocol.**
5. When making revisions to this protocol as requested by the IRB, please follow the instructions outlined in the Study Submission Guide available in the Help Center in CATS IRB (http://irb.psu.edu) for using track changes.

If you need help…	
**University Park and other campuses:**<br>Office for Research Protections Human Research Protection Program<br>The 330 Building, Suite 205<br>University Park, PA 16802-7014<br>Phone: 814-865-1775<br>Fax: 814-863-8699<br>Email: irb-orp@psu.edu	**College of Medicine and Hershey Medical Center:**<br>Human Subjects Protection Office<br>90 Hope Drive, Mail Code A115, P.O. Box 855<br>Hershey, PA 17033<br>(Physical Office Location: Academic Support Building Room 1140)<br>Phone: 717-531-5687<br>Fax number: 717-531-3937<br>Email: irb-hspo@psu.edu

Table of Contents

1.0 Objectives

2.0 Background

3.0 Inclusion and Exclusion Criteria

4.0 Recruitment Methods

5.0 Consent Process and Documentation

6.0 HIPAA Research Authorization and/or Waiver or Alteration of Authorization

7.0 Study Design and Procedures

8.0 Subject Numbers and Statistical Plan

9.0 Confidentiality, Privacy and Data Management

10.0 Data and Safety Monitoring Plan

11.0 Risks

12.0 Potential Benefits to Subjects and Others

13.0 Sharing Results with Subjects

14.0 Subject Stipend (Compensation) and/or Travel Reimbursements

15.0 Economic Burden to Subjects

16.0 Resources Available

17.0 Other Approvals

18.0 Multi-Site Research

19.0 Adverse Event Reporting

20.0 Study Monitoring, Auditing and Inspecting

21.0 Future Undetermined Research: Data and Specimen Banking

22.0 References

1.0 Objectives

1.1 Study Objectives

OBJECTIVE1. Partnering with 14 PA public high schools (letters of commitment obtained for all 14 high schools) serving an estimated 12,582 predominantly minority, rural, urban, and/or low socioeconomic status (SES) students, we will conduct a randomized controlled trial, with student randomization by grade, to compare the effectiveness of universal school-based screening for adolescent major depressive disorder (MDD) versus the existing process of targeted screening based on concerning behavior.

- *Hypothesis 1: Universal screening will increase the number of adolescents with MDD screened, identified and engaged in treatment (MDD composite).*
- *Hypothesis 2 (Moderating): Universal screening will increase the historically poorer rates of MDD screening, identification and treatment engagement for females*
- *Hypothesis 3 (Moderating): Universal screening will increase the historically poorer rates of MDD screening, identification and treatment engagement for rural adolescents*

OBJECTIVE 2. Analyze the impact of MDD screening on secondary outcomes currently collected by the school's Student Assistance Program (SAP) and the school district. These data will be obtained in aggregate by grade level at each participating school (a total of 64 data points for each item; 4 grades x 14 schools, see note below re: PCORI). Please note, secondary outcomes may no longer be available due to COVID-19 school closures and cancellation of state standardized testing.

- *Hypothesis 1. Standardized test scores (e.g. Keystone exams, PSAT, SAT, ACT) will improve in school populations with universal MDD screening secondary to earlier identification, treatment referral and engagement.*
- *Hypothesis 2. Student school policy violations and suspensions will remain unchanged among school populations with the universal MDD screening approach.*

Please note: We recently received funding from the Patient Centered Outcomes Research Institute (PCORI) for a complementary project to the one above. Aims for this HRSA study and the PCORI study have been adjusted with the permission of both organizations to be complementary but distinct. The PCORI project is in contract negotiations but will involve only urban school districts focusing on the effectiveness of MDD screening in minority populations (deleted objective #1, hypothesis #2 above) HRSA will remain focused on rural schools, which are primarily white (hence the rationale for removing objective #1, hypothesis #2). Secondary outcomes have similarly been adjusted to be complementary but distinct from each other. The study will be registered as one RCT in clinical trials.gov again with the approval of the funding organizations. The final analysis will involve both the HRSA and PCORI funded schools.

1.2 Primary Study Endpoints

MDD composite which includes MDD screen positive for the universal screening arm (or concern in the targeted screening group), MDD identification, and MDD treatment engagement. Each of the primary study endpoints will be collected at the individual level by the school district by the end of the school year. No identifiable information will be collected by study staff.

Universal Group

1) Adolescents with PHQ-9 score $\geq$11 (screen Sept-March of the school year) or who at any point in the year exhibit behavior concerning for MDD prompting a SAP triage request, 2) Adolescents identified with MDD by SAP triage, and 3) Adolescents who successfully engage with at least one SAP recommendation

Targeted Group

1) Adolescents with behavior concerning for MDD prompting a SAP triage request, 2) Adolescents identified with MDD by SAP triage, and 3) Adolescents who successfully engage with at least one SAP recommendation

Concern for MDD based on a primary or secondary potentially MDD related SAP "incoming referral reason"

SAP triage is not diagnostic, so MDD identified based on recommendations for MDD related school or community services (e.g. mental health treatment services)

1.3 Secondary Study Endpoints

The secondary endpoints listed below are individual level data points that will be obtained from the school district and/or SAP. No identifiable information will be collected by study staff.

MDD screen positive or MDD concern prompting Student Assistance Program triage

Universal screening arm: Adolescents who have a PHQ-9 score $\geq$11 (screening with the PHQ-9 is planned for September to March) or who at any point in the school year exhibit behavior concerning for MDD which prompts self or collateral request for SAP triage.

Targeted screening arm: Adolescents with behavior concerning for MDD which prompts self or collateral request for SAP triage at any point during the school year.

Suicidal adolescent (includes suicidal thoughts [positive response to PHQ-9 item 9], attempts and completed)

Universal screening arm: Patient health questionnaire positive response to question #9 re: suicidal thoughts, which requires management by the state-mandated school crisis plan or student self or collateral report of suicidal thoughts, which requires management by the state-mandated school crisis plan (source school district).

Targeted screening arm: Student self or collateral report of suicidal thoughts, which requires management by the state-mandated school crisis plan (source school district).
Any student suicide attempts or completed suicides shared with the school district will also be included.

MDD identification

Universal and targeted screening arms: Adolescents who are identified as having MDD based on triage by the school SAP team. As SAP triage is not diagnostic, MDD identified will be based on SAP recommendations for school or community services which are MDD related (e.g. mental health treatment services).

MDD treatment engagement

Universal and targeted screening arms: Adolescents who successfully engage with at least one SAP recommendation. This may be fulfilled by parental report that an appointment was successfully scheduled

The secondary endpoints listed below are aggregate data points by grade level that will be obtained from the school district and/or SAP. No identifiable information will be collected by study staff. Please

note, secondary outcomes may no longer be available due to COVID-19 school closures and cancellation of state standardized testing.

Standardized test scores (i.e. Keystone exams, PSAT, SAT/ACT)
Student school policy violations and/or suspensions
Missed school days
Grade point averages
Grade advancement or graduation rates

Additional data points to be obtained for subgroup analyses include:

- At the individual level:
  - Sex - male or female
  - Ethnicity - Hispanic vs. non-Hispanic
  - Race - white, black or other
  - Urban/rural – students will be categorized as enrolled in urban or rural school districts based on the definition applied by The Center for Rural PA, a Legislative Agency of the PA General Assembly for the district

- At the aggregate level, the following variables will be collected:
  - District socioeconomic status
  - School size – school enrollment. Data will be obtained from school districts based on enrollment as of October 1 of the RCT
  - Ratio of guidance counselors/students – The ratio of counselors to students will be obtained from each school district
  - School-based mental health services – schools will be categorized by the availability of school-based mental health services (yes vs. no) based on information obtained from each school district.

We need to collect data at the individual level for 3 main reasons. First, we expect approximately 20% of students to opt out from the study, with opt out rates varying by grade. Aggregate data would necessarily include outcomes for students who are not enrolled in the study. This is particularly problematic for the universal screening group because students who opt out will not be offered the depression screening tool (PHQ-9). Second, we need to obtain gender and race/ethnicity to conduct important planned secondary analyses (subgroup analyses) that will examine efficacy of universal screening by these groups. In particular, we expect that females and minority students will have much higher rates of major depression disorder identified in the universal screening group. Third, in the universal screening group, we will be able to link responses to the PHQ-9 to outcomes, which will allow for estimation of important measures such as the false positive rate of the PHQ-9 (score ≥11, but SAP process determines no further referrals are needed). These measures will inform decision-making regarding the potential for implementation of the intervention (in other schools) should the results of the trial ultimately show efficacy.

2.0 Background

2.1 Scientific Background and Gaps

The prevalence of annual major depressive disorder (MDD) episodes among US adolescents rose from 8.3% in 2008 to 12.5% in 2015.[1] Close to 30% of adolescents with MDD reported suicidality in the prior year, with more than one in ten making a suicide attempt.[2] As a result, suicide was the 2nd leading cause of death among youth 10-24 years of age as of 2014.[3] Baseline data from HealthyPeople.gov

found that only 2.1% of adolescent primary care office visits included depression screening in the years 2005-2007.[4] Inequalities were reported for women who are three times more likely to have MDD, but less likely to be treated than males.[1,2,6] In response to the growing mental health crisis, the US Preventive Services Task Force (USPSTF) endorsed universal screening for adolescent MDD in primary care in 2009.[7,8] The HealthyPeople.gov 2020 goal is a 10% increase in screening to a rate of 2.3%, which fails to address this adolescent public health crisis.[4] The USPSTF universal MDD screening recommendation was based on evidence that treatment of MDD is associated with moderate benefit.[7,8] While most experts in family medicine, pediatrics, psychology and child psychiatry agree that surveillance of adolescents at high-risk for MDD is warranted, the USPSTF updated their recommendations in 2016 with a call to address several knowledge gaps:

1) Does screening increase the proportion of adolescents identified with MDD?

2) What are the benefits and unintended consequences of MDD screening for subgroups: age, sex, race, ethnicity and socioeconomic status (SES)?

3) What are the benefits and unintended consequences of screening in nonclinical settings?[8]

We propose that schools may provide an effective setting to conduct universal MDD screening. While over half of US adolescents do not have annual preventive health visits most attend school.[9] The regular contact with schools compared to contact with the medical setting has been used to advocate for many school-based universal health screenings that impact academic success (e.g. vision, hearing). However, while current school screenings address multiple physical health domains, none address mental health.[10] Targeted mental health screening is the current school process for students who display signs concerning for MDD and results in referral to the school's Student Assistance Program (SAP). SAP operates in all 500 Pennsylvania school districts and functions similar to a triage service by assessing symptom severity, and then if appropriate, providing referrals to school or community-based mental health resources.[11] Students may self-refer, but all other SAP referrals depend upon a student exhibiting concerning behavior that is detected by school staff, peers or parents, which results in a targeted screening process with obvious limitations.

2.2 Previous Data

Our research team is acutely aware of the concerns the topic of adolescent depression screening may raise among school staff, providers, parents and adolescents. We have had ample opportunity in our pilot work to discuss and address many of these issues as outlined below. First, from April-Sept 2016 we conducted eight focus groups (7-10 participants each, n=62) to better understand the perspective of key stakeholders regarding Whole Child Health, specifically the importance of both physical and mental wellness. These focus groups included 2 parent groups, 2 school nurse groups, 2 groups of school teachers and administrators and 2 groups of medical providers (pediatrics and family medicine). The work was funded by the Penn State Social Science Research Institute-Children, Youth and Families Consortium. Participant conversations were instrumental in shaping the current proposal. In addition, following the aforementioned focus groups, we conducted a Community Engagement Studio in August 2017 funded by the Penn State Center for Translational Science Institute. These 2 hour sessions are specifically intended to inform grant proposals that depend upon successful community engagement. Participant perspectives ranged from adolescent to parent, school staff, the leaders of two mental health and suicide prevention organizations (Aevidum and the Jana Marie Foundation), a Behavioral Health Managed Care company representative and the project director of Pennsylvania's Garrett Lee Smith Youth Suicide Prevention Grant in addition to our Penn State Research team.

2.3 Study Rationale

Rates of major depression are rising among US adolescents paralleled by a rise in the rate of adolescent suicides. The most recent data indicates that 1 in 8 adolescents (12.5%) experienced an MDD episode in the past 12 months.[1] The most striking increase in MDD trends was for females across all racial and ethnic groups. Adolescent females demonstrated rates of MDD episodes over 3 times that of males

(19.5% vs. 5.8%).[1,6] Some authors have suggested that adolescent females have increased exposure to depression risk factors including cyberbullying, mobile phone use and texting.[6] Along with the rise in MDD episodes, adolescent females have demonstrated a significant rise in emergency department visits for nonfatal self-inflicted injuries with rates since 2009 increasing by 19% annually from 110 in 2009 to 318 per 100,000 in 2015.[12] Self-inflicted injury is one of the strongest risk factors for suicide, and the suicide rate for female adolescents reached its highest in the past 40 years according to 2017 Centers for Disease Control and Prevention data.[13,14]

Adolescent MDD has negative effects on academic performance, with increasing severity of depressive symptoms linked to a lower grade point average as well as subjective assessments of increased school workload and concentration difficulties. Adolescents with untreated MDD experience poorer interpersonal relationships, lower self-esteem, social isolation, and increased risk-taking behaviors including substance use, as well as multiple physical and mental health comorbidities in adulthood.[7,8,15-17] For 60-90% of adolescents, symptoms of a MDD episode may remit within in a year. The larger problem is that 50-75% of these adolescents will develop subsequent MDD episodes within 5 years, resulting a chronic or relapsing disorder.[18] Studies also suggest that recovery is not complete between episodes, with most individuals reporting residual symptoms or impairment.[18]

Despite the rising rates of depression, there has been no change in mental health treatment among adolescents with a MDD episode from 2005-2014.[6] Only 36-44% of children and adolescents with MDD receive treatment, underscoring that MDD is underdiagnosed and undertreated.[6] This disparity is especially pronounced for disadvantaged populations. Even for those who have a primary care provider, data from HealthyPeople.gov indicates a steady decline in rates of screening with only 1.4% of primary care office visits including MDD screening as of 2009- 2011.[4] In addition to minorities, rural youth and those of lower SES receive fewer preventive care services than their white, urban, high SES counterparts, further limiting their access to MDD screening in the context of well-care.[20,21]

3.0 Inclusion and Exclusion Criteria

3.1 Inclusion Criteria

Students in grades 9-12 at 14 public schools [redacted] in Pennsylvania that previously committed to partner with us in this project. HRP-504- School Permission to Conduct Research forms will be uploaded in our CATS application documentation for each participating district to show district approval of the opt out procedure and to agree with their 3rd Party Protection of Pupil Rights Amendment (PPRA) policies. Further, included as supporting documents are letters from [redacted] who have all acknowledged and shown support of the opt out approach.

School staff who assisted with the screener/screening process will be asked to complete a 45 minute interview using our Year 1 or Year 2 feedback guide documents (included in supporting documents).

3.2 Exclusion Criteria

Students whose parents complete the opt-out consent
Students not enrolled in one of the participating schools
Students not in grades 9-12
Students with disabilities that are deemed unable to participate by the school district

3.3 Early Withdrawal of Subjects

3.3.1 Criteria for removal from study

N/A

3.3.2 Follow-up for withdrawn subjects

N/A

4.0 Recruitment Methods

4.1 Identification of subjects

Three PA public schools committed to partner with us to complete the proposed work in the 2018-2019 school year. Another 6 rural schools will participate in the 2019-2020 school year and this information will be submitted to the IRB in advance of the actual screening. PI [redacted] and co-investigator [redacted] have previously partnered with several of these school districts through their prior research. In total, the rural schools serve approximately 3,900 students (HRSA Study) and were selected as they represent a large number of low SES, rural student populations who have known disparities in mental health services. The additional PCORI funded urban schools [redacted] adds an additional 8,150 students. Additionally, we will recruit 1-2 school staff members to participate in our feedback guide interview upon completion of the screener/screening process year. We are not collecting any PHI, nor will names of the school staff participants be disclosed in the use of manuscripts/ written publications.

4.2 Recruitment process

After discussion with our stakeholders and the Penn State Institutional Review Board, we will pursue opt-out consent for screening given the importance of MDD screening and the low-risk aside from identification of a suicidal student. Parents will be informed of their child's enrollment in screening and given the opportunity to opt out prior to the fall intervention. For this proposal, in cases of a shared custody agreement, if either parent or guardian opts out of the study, the student will be considered ineligible for enrollment. In this case, no information will be collected, even in the case of students randomized to the targeted screening arm, which is the usual school process. An additional opt-out letter was created for HRSA schools, as one participating school did not send opt-out letters to parents whose children were randomized to the target (control) arm of the trial. This letter has been included in supporting documents and will be sent in spring 2020, after the intervention has ended. No information will be collected for those students whose parents return these opt-out forms by the end of the school year. Also, any participating student randomized to the universal screening arm who does not assent at the time of screening will not be required to complete the screening form. Students 18 years and older are anticipated to be a small minority of the students at the start of the academic year, but they will also have the opportunity to opt out of study involvement if desired. We will be contacting our primary contacts from each of our participating districts to obtain implied/verbal consent for participating in our feedback guide interviews.

4.3 Recruitment materials

No recruitment materials will be needed for this study. However, a letter will be sent home to parents to inform them of their child's enrollment in the screening and given the opportunity to opt out prior to the intervention. If they do not wish to participate, parents will be asked to return the Opt-Out form in-person or by mail. Opt-out letters will be printed on district letterhead to include the participating school's mailing address. Parents may also return the form signed and scanned, if the school decides to send the letter via email. A copy of the PHQ-9 questionnaire will be included in the letter sent to the parents. Additionally, the opt-out letter will be translated into a Spanish by the Department of Care Coordination and provided to each school. An additional opt-out letter was created for HRSA schools, as one participating school did not send opt-out letters to parents whose children were randomized to the target (control) arm of the trial. This letter has been included in supporting documents and will be sent in spring 2020, after the intervention has ended. Parents will be asked to email or mail their signed opt-out letter if they do not want their child to participate in the control arm.

4.4 Eligibility/screening of subjects

N/A

5.0 Consent Process and Documentation

5.1 Consent Process

5.1.1 Obtaining Informed Consent

5.1.1.1 Timing and Location of Consent

N/A

5.1.1.2 Coercion or Undue Influence during Consent

N/A

5.1.2 Waiver or alteration of the informed consent requirement

We are requesting a waiver of the informed consent requirement. We are pursuing opt-out consent for screening given the important of MDD screening and the low-risk aside from identification of a suicidal student. At 14 years of age, PA youth are eligible to consent to mental health services without parental consent. Our use of the opt out is really intended to include and engage the parents and communities we are working with. The opt out will be a letter sent home to parents (either via email or regular mail as per school preference) giving them the option to decline participation for their student. Schools will be responsible for tracking students whose parents have opted-out. In addition, students in the universal arm will have the option to decline participation themselves on the screening day via the iPad handed to them. The first screen will describe PHQ-9; inform students that participation is voluntary; and participation may be stopped at any time and will not affect their school standing or grades.

We are requesting a waiver of informed consent by utilizing the HRP-585- HSPO Summary Explanation Research document for each of the staff members chosen from our participating districts to complete our interview using the Final or Year 2 Feedback Guide Interview documents.

5.2 Consent Documentation

5.2.1 Written Documentation of Consent

N/A

5.2.2 Waiver of Documentation of Consent (Implied consent, Verbal consent, etc.)

A summary explanation will be used to inform participants about the study allowing them to choose to participate in the study. They will provide us verbal consent. Staff members from two schools completed the Final Feedback Guide interview [redacted] as a pilot to ensure the final version of the Feedback Guide was thorough and comprehensive. These staff members will receive the approved summary explanation, along with a copy of their completed feedback guide to ensure final consent. To better understand the feasibility of the screening process, study staff created a Year 2 feedback guide as a follow-up. A summary explanation for the Year 2 feedback guide will be used to inform participants of the study before participating in the Year 2 Feedback guide. This document has been uploaded to the supporting documents section.

5.3 Consent – Other Considerations

5.3.1 Non-English Speaking Subjects

A Spanish translator was included in the grant and will join the research team for the MDD screening at schools where these services would be needed. In addition, the opt out information will be translated and sent in Spanish for those parents who the school indicates would have trouble with the English version forms. The assent will also be translated into Spanish for those students who indicate that Spanish is their preferred language. The PHQ-9 is already available in multiple languages including Spanish.

5.3.2 Cognitively Impaired Adults

N/A

5.3.2.1 Capability of Providing Consent

N/A

5.3.2.2 Adults Unable To Consent

N/A

5.3.2.3 Assent of Adults Unable to Consent

N/A

5.3.3 Subjects who are not yet adults (infants, children, teenagers)

5.3.3.1 Parental Permission

We are requesting a waiver of informed consent. Rather, parents/guardians will receive an opt-out form. We are pursuing opt-out consent for screening given the important of MDD screening and the low-risk aside from identification of a suicidal student. At 14 years of age, PA youth are eligible to consent to mental health services without parental consent. Our use of the opt out is really intended to include and engage the parents and communities we are working with.

5.3.3.2 Assent of subjects who are not yet adults

Students in the universal arm will have the option to decline participation themselves on the screening day via the iPad handed to them. The first screen will describe PHQ-9; inform students that participation is voluntary; and participation may be stopped at any time and will not affect their school standing. The completion of the PHQ-9 implies a student's voluntary consent to participate in the research. Students who decide not to participate will not complete the PHQ-9, but will still be tracked similar to students randomized to the targeted screening arm. The study team will obtain their demographic information and the student will be followed through the academic year for SAP triage intakes initiated by the standard pathway (concern by teachers, nurse, parent, peer, or self-referral), any referrals and treatment engagement. No identifiable information will be obtained and it will be noted in study records that the student did not assent to participate in the MDD screener. A copy of this assent form is included in the consent form section.

6.0 HIPAA Research Authorization and/or Waiver or Alteration of Authorization

6.1 Authorization and/or Waiver or Alteration of Authorization for the Uses and Disclosures of PHI

Check all that apply:

☒ **Not applicable, no identifiable protected health information (PHI) is accessed, used or disclosed in this study.** *[Mark all parts of sections 6.2 and 6.3 as not applicable]*

☐ **Authorization will be obtained and documented as part of the consent process.** *[If this is the only box checked, mark sections 6.2 and 6.3 as not applicable]*

☐ **Partial waiver is requested for recruitment purposes only (Check this box if patients' medical records will be accessed to determine eligibility before consent/authorization has been obtained).** *[Complete all parts of sections 6.2 and 6.3]*

☐ **Full waiver is requested for entire research study (e.g., medical record review studies).** *[Complete all parts of sections 6.2 and 6.3]*

☐ **Alteration is requested to waive requirement for written documentation of authorization (verbal authorization will be obtained).** *[Complete all parts of sections 6.2 and 6.3]*

6.2 Waiver or Alteration of Authorization for the Uses and Disclosures of PHI

6.2.1 Access, use or disclosure of PHI representing no more than a minimal risk to the privacy of the individual

6.2.1.1 Plan to protect PHI from improper use or disclosure
N/A

6.2.1.2 Plan to destroy identifiers or a justification for retaining identifiers
N/A

6.2.2 Explanation for why the research could not practicably be conducted without access to and use of PHI
N/A

6.2.3 Explanation for why the research could not practicably be conducted without the waiver or alteration of authorization
N/A

6.3 Waiver or alteration of authorization statements of agreement
N/A

7.0 Study Design and Procedures

7.1 Study Design
Due to the timeline for this funding opportunity, with an anticipated July 2018 start, 3 schools will engage in the randomized control trial (RCT) in study year 1 and the additional 13 schools in study year 2. Many large scale randomized control trials do not enroll all participants at one time point, and in many cases enrollment occurs slowly over the course of several years. We do not anticipate there will be major changes over the course of two academic years that would significantly alter the results compared to conducting the RCT in the same academic year, especially because students will be randomized within schools. Finally, staggering enrollment will give the research team the opportunity to troubleshoot any unanticipated issues with the first 3 participating schools. The intervention ended early in study year 2 (March 2020) due to the COVID-19 pandemic and schools subsequently ending in-person instruction.

The study team was unable to complete PHQ-9 screeners with two participating schools [redacted]. SAP outcome data will still be collected for all enrolled students (i.e., those whose parents did not return the opt-out form) for the 2019-2020 (study year 2) school year.
At the completion of the year one screener/ screening process, school staff will be asked to complete the Final Feedback Guide document to assist in our research/ knowledge of the study process and procedures. Staff members from two schools completed the Feedback Guide interview [redacted] as a pilot to ensure the final version of the Feedback Guide was thorough and comprehensive.
At the completion of the of the 2019/2020 school year, the 3 schools that participated in the RCT during the 2018/2019 school year, will be asked to complete the Year 2 feedback guide interview document to assist in our research/knowledge of the long-term feasibility and sustainability of study processes and procedures. During their second year of study participation, school participants will be asked to complete the feedback guide interview with research personnel. Due to the COVID-19 pandemic occurring during the 2019-2020 school year, we have added questions relevant to student mental health concerns.
The remaining 13 schools who participated in the RCT during the 2019/2020 school year will be asked to go through the Final Feedback guide. Research personnel will contact school staff and go through the Final Feedback guide at the end of the 2019/2020 school year. Due to the COVID-19 pandemic occurring during the 2019/2020 school year, we have updated the Final Feedback guide to include questions relevant to student mental health concerns.

7.2 Study Procedures

7.2.1 Enrollment

Parents will be informed of their child's enrollment in screening and given the opportunity to opt out prior to the fall intervention. The opt out will be a letter sent home to parents (either via email or regular mail as per school preference) giving them the option to decline participation for their student. The study team will provide each school with unique study IDs to be assigned to every student, grades 9-12. Study IDs will include 8 numbers, the first two representing the school, the second two the grade and the remaining 4 will be unique to each participating student. Schools will be required to assign these unique study IDs to each student, grades 9-12. Schools will complete a linking list (spreadsheet template) ensuring that all students, grades 9-12 are included. A completed linking list will include student names (first and last), PASECURED ID, unique study ID and demographics information (grade, age, sex, race and ethnicity). Schools will remove student names and PASECUREID before sending the spreadsheet to the study team. *The full linking list (including student name and PASECURIEDID) will remain on the school's spreadsheet and in the school's possession for tracking of study outcomes through the year.* By using a unique study ID for each student, the study team will never receive identifiable information of students. If a parent returns the opt-out form, school staff will still assign a student a unique study ID but no demographic information will be included. This is so the study team may properly report the number of opt-out letters returned. This procedure ensures that students whose parents have opted-out are not tracked throughout the year.

At the time of the actual universal screening students will also be provided the chance to opt out by clicking the appropriate opt out box on the iPad handed to them. We anticipate this will lead to study enrollment of 80% of eligible students. The first screen will describe PHQ-9 and that proceeding is voluntary. The completion of the PHQ-9 implies a student's voluntary consent to participate in the research. Unless the parent opt out is returned, participants in both arms will be followed through the school year for SAP triage, follow-up referrals and treatment engagement. Those students in the universal screening arm with PHQ-9 scores $\geq$ 11 (MDD screen positive) corresponding to moderate depressive symptoms, will proceed through the standard process for anyone referred by traditional means to a SAP triage interview. The student will either be referred to appropriate community or

school-based treatment or SAP will determine no follow-up is needed. For those who are recommended to additional services by SAP, treatment engagement will be tracked per current SAP processes. The study team will receive individual level outcome data from the school district, containing no identifiable information.

Additionally, we will recruit 1-2 school staff members to participate in our feedback guide interview upon completion of the screener/screening process year. We are not collecting any PHI, nor will names of the school staff participants be disclosed in the use of manuscripts/ written publications. Staff members from two schools were recruited and completed the Final Feedback Guide interview [redacted] as a pilot to ensure the final version of the Feedback Guide was thorough and comprehensive.

To better understand the long-term feasibility of the screening process, school contacts who participated in the first year of screening will be asked to participate in a Year 2 feedback guide interview. This guide will provide information about the screening process impacts and sustainability in [redacted] high schools. The remaining schools who participated in screenings during the 2019-2020 school year, will participate in the Year 2 feedback guide interview at the end of the 2020-2021 school year.

7.2.2 Randomization

We will randomize by grade levels within each school to receive either one-time universal screening (via PHQ-9) or targeted screening (current SAP process). For the schools included in the study, half of the schools (50%) will be randomized such that students in 9th and 11th grades will receive universal screening and students in 10th and 12th grades will receive targeted screening, and the other half (50%) will be randomized such that students in 9th and 11th grades will receive targeted screening and students in 10th and 12th grades will receive universal screening. Randomization will be done only for students whose parents do not signal an unwillingness to participate in the study ("opt out"). Students and study personnel will not be blinded to randomized group at each school site. Randomization will be done by grade level primarily for pragmatic reasons because many PA health-based screenings are grade-specific (e.g. hearing screen in 11th grade) and screenings for a grade occur at the same time. The study will be conducted within schools, but it is not a cluster randomized study, in which an entire school (cluster) is assigned to one of the study groups. A cluster randomized study was considered but ultimately not pursued because randomization within schools controls for (1) within-school (community) factors that may contribute to higher or lower rates of SAP referral, (2) differences in school sizes, and (3) potential differences in rates of parental opt out among schools. These benefits were balanced against the concern of potential contamination between study groups, whereby those in the targeted screening group may benefit from the school-wide push to conduct universal screening.

7.2.3 Universal MDD Screening Arm (Intervention – Treatment)

Students randomized to the universal screening arm will complete a PHQ-9. This screening tool includes nine close-ended questions with a scoring system ranging from 0 to 27. The PHQ-9 screens will be administered on an iPad with an internet connection which allows direct entry of the results in REDCap. To prevent the duplication of unique study IDs, the study team will ensure all students have been assigned a unique study identification number prior to the screening. A list of participant IDs will be provided to the school member present during the screening. Once the unique study ID has been entered into REDCap, the study staff will give the iPad to the student to assent and complete the PHQ-9. This process will ensure that the correct study ID is used and prevent duplication of study IDs. Further, this will safeguard against any student from participating whose parent returned the opt-out letter. No names or other identifying information will be used. Paper copies of the assent and PHQ-9 will be

available as a backup should problems arise with internet connectivity. The same measures will be taken to ensure no names or other identifying information is used or collected.

In order to immediately identify suicidal intent the survey will be set to flag positive questions in real time. When students have completed the PHQ-9 screen the REDCap survey will prompt them to hand the completed questionnaire to study staff, who will see a screen indicating a positive flag. Students with suicidal intent (question #9 any response besides "Not at all") will receive immediate evaluation and referral to emergency care as per current school protocols. PA schools are required to have a plan to address suicidal students (Act 71). A suicidal participant identified during the screening would not be allowed to leave the screening area unless accompanied by appropriate school or research staff. This student would then proceed through the standard school pathway for managing a student with intentions of self-harm. To ensure school staff is comfortable to manage a student in crisis and that this persists beyond the period of the grant, at least 5 staff per school in addition to at least 4 Penn State research staff will complete online evidence-based suicide prevention training (Question, Persuade, and Refer [QPR] Suicide Triage Training). In addition, all school crisis plans will be carefully reviewed with staff following the training to ensure the steps are realistic and staff is comfortable to execute the plan. The district identified, QPR trained staff member will be available at the time of screening. Study staff who are also QPR trained, will be present during the screening.

7.2.4 Targeted Screening Arm (Current Process – Control)

Students randomized to the targeted screening arm will complete their routine school-based screenings. Students will be followed through the academic year for SAP triage intakes initiated by the standard pathway (concern by teachers, nurse, parent, peer, or self-referral), any referrals and treatment engagement.

7.2.5 Sharing Screening Results

At 14 years of age, PA youth are eligible to consent to mental health services without parental consent, therefore, screening results will not be shared with parents. Those students with scores $\geq$ 11 (MDD screen positive) corresponding to moderate depressive symptoms will, however, proceed through the standard process for anyone referred by traditional means to a SAP triage interview. The student will either refer to appropriate community or school-based treatment or therapy (MDD identified) or SAP will determine no follow-up is needed. For those who are recommended to additional services by SAP, treatment engagement will be tracked per current SAP process. As per current school policy, students with suicidal feelings will receive immediate referral to emergency care and parents will be notified by the school.

7.3 Duration of Participation

The student's participation is limited to the 5 minutes it takes for them to participate in the screening process.

The school staff members who participate in the feedback guide interview is limited to the 45 minutes it takes for them to complete it.

8.0 Subject Numbers and Statistical Plan

8.1 Number of Subjects

The 9 HRSA funded schools to be included in the study have an estimated total enrollment of approximately 3,900 students (the PCORI funded urban schools have a total enrollment of approximately 8,150 students). The overall rate of parental opt out is expected to be around 20%,

resulting in approximately 9,640 students included in the study. We assumed a 15% attrition rate for students who move, drop out of school, or opt out later in the school year. This number will be finalized once we have a final confirmed list of participating schools. However, as the total number of students is greater than that originally projected below, the sample size determination section will be unchanged.

8.2 Sample size determination

A total of 4,820 (estimated) students in each randomized group (an overall sample size of 9,640 [estimated]) yields >99% statistical power to detect a difference of 3% versus 6% using a 2-sided test conducted at a Type I error rate of 5% in a mixed effect logistic regression model.

8.3 Statistical methods

The principles of intention-to-treat (ITT) will be used for all statistical analyses related to primary and secondary aims. For the primary aim comparing universal to targeted screening, the statistical analysis will be conducted using a mixed effects logistic regression model. The primary outcome, MDD composite, will be an indicator whether a student was screen positive (or concerns raised in the targeted screening arm), identified as having MDD and subsequently engaged in treatment (1=yes, 0=no). The model will include a fixed effect for randomized group (0=targeted screening, 1=universal screening) and a random effect for school. The random effect accounts for correlation among students enrolled within the same school. The primary parameter of interest will be the log odds of MDD composite in the universal screening group compared to the targeted screening group. Statistical significance of the log odds will be assessed using a 2-sided Wald test. Point estimates for the odds ratio along with a 95% confidence interval will be reported.

For the analysis of Hypotheses 2 and 3 (Objective 1) evaluating universal screening and targeted screening by selected subgroups, the same mixed effects logistic regression modeling framework will be used, but the model will be extended by including a fixed effect for subgroup and an interaction effect for subgroup by randomized group. The interaction term will be the parameter of interest. A significant interaction term indicates that rates of MDD treatment engagement for universal versus targeted screening differ by subgroup level (e.g. female vs. male). For the secondary analyses (Objective 2) that evaluates universal screening and targeted screening based on school SAP data, we will have only 64 total data points (4 grades in each of the 14 schools). Mixed-effects linear (continuous outcomes) and logistic regression (binary outcomes) will be used, as appropriate, with a fixed effect for randomized group and a random effect for school. The parameter of interest will be the log odds for the universal compared to the targeted screening group. Due to the smaller sample size used for these outcomes, these analyses will be considered to be primarily hypothesis-generating. Potential mediating variables (socioeconomic status, ratio of guidance counselors to students and availability of school-based mental health services) will be evaluated for both the Objective 1 and 2 hypotheses.

Additional secondary outcomes will include MDD screen results, MDD concern prompting Student Assistance Program triage, MDD identification and MDD treatment engagement analyzed individually (rather than as part of MDD composite). Finally any suicidal adolescents (suicidal thoughts [positive response to PHQ-9 item 9], attempts and completed) will be analyzed as a secondary outcome.

We need to collect data for individuals for 3 main reasons. First, we expect approximately 20% of students to opt out from the study, with opt out rates varying by grade. Aggregate data would necessarily include outcomes for students who are not enrolled in the study. This is particularly problematic for the universal screening group because students who opt out will not be offered the depression screening tool (PHQ-9). Second, we need to obtain gender and race/ethnicity to conduct important planned secondary analyses (subgroup analyses) that will examine efficacy of universal screening by these groups. In particular, we expect that females and minority students will have much higher rates of major depression disorder identified in the universal screening group. Third, in the

universal screening group, we will be able to link responses to the PHQ-9 to outcomes, which will allow for estimation of important measures such as the false positive rate of the PHQ-9 (score ≥11, but SAP process determines no further referrals are needed). These measures will inform decision-making regarding the potential for implementation of the intervention (in other schools) should the results of the trial ultimately show efficacy.

Efforts will be made to ensure completeness of data where possible, but missing data will occur for a number of anticipated reasons. First, a student may move during the course of the school year to another school district or drop out from school entirely. No data will be collected after this time point. Second, parents may opt out their child from the study at any time during the school year. Third, students who turn 18 during the school may decide to opt out of the study themselves. In both of these instances, data from SAP referrals that occur after opting out will not be collected for purposes of the study. Fourth, during the one-time universal screening phase, students may decide to leave data forms incomplete, including the PHQ-9. To decrease these instances, the PHQ-9 will be taken on an iPad program and the survey will alert if the form is left incomplete.

9.0 Confidentiality, Privacy and Data Management

Please see HRP-598 - Research Data Plan Review Form

10.0 Data and Safety Monitoring Plan

N/A: This study does not involve more than minimal risk to subjects, and the magnitude of harm/discomfort is not greater than that ordinarily encountered in daily life.

11.0 Risks

Risk involved in participating in this study are low aside from identification of a suicidal student, in which case measures are already currently in place in each school building to address.

12.0 Potential Benefits to Subjects and Others

12.1 Potential Benefits to Subjects

Potential benefit to subjects of positive screenings include a referral to SAP to receive support in managing their MDD.

12.2 Potential Benefits to Others

The potential public health impact of the proposed project cannot be overstated. MDD is a prevalent, disabling and a growing US public health problem. The problem is identified by national organizations focused on our country’s health care priorities (Healthy People 2020, US Department of Health and Human Services). MDD leads both to functional impairment and higher rates of morbidity and mortality. In addition, MDD leads to significant social and economic consequences, including increased use of health resources and lost work productivity. A public health goal should include identification of those at risk for depression with the delivery of interventions to these individuals. Schools are a point of intervention with a high potential for early identification and prevention. Currently, fewer than 2 out of every 100 adolescents receives guideline-concordant major depression screening. We propose reaching nearly 80 of every 100 adolescents in the school setting, vastly increasing the identification of adolescents suffering from MDD with a goal to decrease both morbidity and mortality. Schools are an ideal partner for this approach, given their tremendous reach across the nation to nearly all adolescents.

13.0 Sharing Results with Subjects

Individual results will not be shared with other participants.

14.0 Subject Stipend (Compensation) and/or Travel Reimbursements

N/A

15.0 Economic Burden to Subjects

15.1 Costs

There are no financial costs associated with participating in this research.

15.2 Compensation for research-related injury

N/A

16.0 Resources Available

16.1 Facilities and locations

Screening will take place in each of the 4 school buildings previously identified for the 2018-2019 school year. The study team will submit a finalized list of schools in advance of the 2019-2020 screenings.

16.2 Feasibility of recruiting the required number of subjects

All 4 school districts for the 2018-19 year have already expressed interest in participating as evidenced through letters of support. Current relationships through past and present programming with the school districts created feasibility for recruitment.

16.3 PI Time devoted to conducting the research

[redacted] will monitor the progress of the study during all phases and hold bi-weekly meetings with research staff.

16.4 Availability of medical or psychological resources

It is not anticipated that medical or psychological resources will be needed on site, given that study procedures are minimal risk. However, students with suicidal intent will receive immediate evaluation and referral to emergency care as per current school protocols. PA schools are required to have a plan to address suicidal students. A QPR trained staff member will be available at the time of screening. Students with a PHQ-9 score $\geq$11 will proceed to Student Assistance Program triage as per the standard of care by which students exhibiting concerning behavior (outbursts, declining grades) would be referred for assessment.

16.5 Process for informing Study Team

The investigators and project coordinator/study staff have completed their required Collaborative IRB Training Initiative (CITI) in the protection of human research subjects. The study team will be educated on the importance of confidentiality, and proper data handling and storage. Four study team members will also complete the Question, Persuade, Refer suicide triage training in order to assist the school staff as needed during the time of actual screening.

17.0 Other Approvals

17.1 Other Approvals from External Entities

N/A

17.2 Internal PSU Committee Approvals

Check all that apply:

- ☐ Anatomic Pathology – Hershey only – Research involves the collection of tissues or use of pathologic specimens. Upload a copy of HRP-902 - Human Tissue For Research Form on the "Supporting Documents" page in CATS IRB. This form is available in the CATS IRB Library.

- ☐ Animal Care and Use – All campuses – Human research involves animals and humans or the use of human tissues in animals

- ☐ Biosafety – All campuses – Research involves biohazardous materials (human biological specimens in a PSU research lab, biological toxins, carcinogens, infectious agents, recombinant viruses or DNA or gene therapy).

- ☐ Clinical Laboratories – Hershey only – Collection, processing and/or storage of extra tubes of body fluid specimens for research purposes by the Clinical Laboratories; and/or use of body fluids that had been collected for clinical purposes, but are no longer needed for clinical use. Upload a copy of HRP-901 - Human Body Fluids for Research Form on the "Supporting Documents" page in CATS IRB. This form is available in the CATS IRB Library.

- ☐ Clinical Research Center (CRC) Advisory Committee – All campuses – Research involves the use of CRC services in any way.

- ☐ Conflict of Interest Review – All campuses – Research has one or more of study team members indicated as having a financial interest.

- ☐ Radiation Safety – Hershey only – Research involves research-related radiation procedures. All research involving radiation procedures (standard of care and/or research-related) must upload a copy of HRP-903 - Radiation Review Form on the "Supporting Documents" page in CATS IRB. This form is available in the CATS IRB Library.

- ☐ IND/IDE Audit – All campuses – Research in which the PSU researcher holds the IND or IDE or intends to hold the IND or IDE.

- ☒ Scientific Review – Hershey only – All investigator-written research studies requiring review by the convened IRB must provide documentation of scientific review with the IRB submission. The scientific review requirement may be fulfilled by one of the following: (1) external peer-review process; (2) department/institute scientific review committee; or (3) scientific review by the Clinical Research Center Advisory committee. NOTE: Review by the Penn State Hershey Cancer Institute Scientific Review Committee is required if the study involves cancer prevention studies or cancer patients, records and/or tissues. For more information about this requirement see the IRB website at: http://www.pennstatehershey.org/web/irb/home/resources/investigator

18.0 Multi-Site Research

N/A

19.0 Adverse Event Reporting

19.1 Reporting Adverse Reactions and Unanticipated Problems to the Responsible IRB

In accordance with applicable policies of The Pennsylvania State University Institutional Review Board (IRB), the investigator will report, to the IRB, any observed or reported harm (adverse event) experienced by a subject or other individual, which in the opinion of the investigator is determined to be (1) unexpected; and (2) probably related to the research procedures. Harms (adverse events) will be submitted to the IRB in accordance with the IRB policies and procedures.

20.0 Study Monitoring, Auditing and Inspecting

20.1 Auditing and Inspecting

The investigator will permit study-related monitoring, audits, and inspections by the Penn State quality assurance program office(s), IRB, the sponsor, and government regulatory bodies, of all study related documents (e.g., source documents, regulatory documents, data collection instruments, study data etc.). The investigator will ensure the capability for inspections of applicable study-related facilities (e.g., pharmacy, diagnostic laboratory, etc.).

21.0 Future Undetermined Research: Data and Specimen Banking

N/A

21.1 Data and/or specimens being stored

N/A

21.2 Location of storage

N/A

21.3 Duration of storage

N/A

21.4 Access to data and/or specimens

N/A

21.5 Procedures to release data or specimens

N/A

21.6 Process for returning results

N/A

22.0 References

1. Office of Disease Prevention and Health Promotion. Healthy People.gov. Mental Health and Mental Disorders. MNMD-4.1 Reduce the proportion of adoelscents aged 12 to 17 years who experience major depressive episodes. https://www.healthypeople.gov/2020/data-search/Search-the-Data#objid=4813; Updated

December 11, 2017. Accessed December 11, 2017.
2. Avenevoli S, Swendsen J, He JP, Burstein M, Merikangas KR. Major depression in the national comorbidity survey-adolescent supplement: prevalence, correlates, and treatment. *J Am Acad Child Adolesc Psychiatry.* 2015;54(1):37-44.e2.
3. Heron M. Deaths: Leading causes for 2014. National Vital Statistics Reports. 2016;65(5). Hyattsville, MD: National Center for Health Statistics. https://www.cdc.gov/nchs/data/nvsr/nvsr65/nvsr65_05.pdf. Accessed January 3, 2018.
4. Office of Disease Prevention and Health Promotion. Healthy People.gov. Mental Health and Mental Disorders. MHMD-11.2. Increase the proportion of primary care physician office visits where youth aged 12 to 18 years are screened for depression. https://www.healthypeople.gov/2020/data-search/Search-the-Data#objid=4808; Updated December 11, 2017. Accessed December 11, 2017.
5. Zenlea IS, Milliren CE, Mednick L, Rhodes ET. Depression screening in adolescents in the United States: a national study of ambulatory office-based practice. *Acad Pediatr.* 2014;14(2):186-191.
6. Mojtabai R, Olfson M, Han B. National trends in the prevalence and treatment of depression in adolescents and young adults. *Pediatrics.* 2016;138(6):e20161878.
7. Forman-Hoffman V, McClure E, McKeeman J, et al. Screening for major depressive disorder in children and adolescents: A systematic review for the U.S. Preventive Services Task Force. *Ann Intern Med.* 2016;164(5):342-349.
8. Siu AL, US Preventive Services Task Force. Screening for depression in children and adolescents: U.S. Preventive Services Task Force recommendation statement. *Ann Internal Med.* 2016;164(5):360-366.
9. Adams SH, Park MJ, Twietmeyer L, Brindis CD, Irwin CE, Jr. Association Between Adolescent Preventive Care and the Role of the Affordable Care Act. *JAMA Pediatr.* 2017 Nov 6. doi: 10.1001/jamapediatrics.2017.3140.
10. Pennsylvania House of Representatives. House Co-Sponsorship Memoranda. House of Representatives Session of 2015-2016 Regular Session. http://www.legis.state.pa.us/cfdocs/Legis/CSM/showMemoPublic.cfm?chamber=H&SPick=20150&cosponId=20186. Published May 6, 2016. Accessed January 3, 2018.
11. Student Assistance Program FY 2015-2016. Mental Health-Drug and Alcohol Statistical Data. https://sapjqrs.org/uploadedfiles/JQRS%20Exec%20Summary%202015-16.pdf. Accessed December 11, 2017.
12. Mercado MC, Holland K, Leemis RW, Stone DM, Wang J. Trends in Emergency Department Visits for Nonfatal Self-inflicted Injuries Among Youth Aged 10 to 24 Years in the United States, 2001-2015. *JAMA.*2017;318(19):1931-1933.
13. Centers for Disease Control and Prevention. QuickStats: Suicide Rates for Teens Aged 15-19 Years, by Sex – United States , 1975-2015. *MMWR Wkly Rep* 2017; 66(30):816. https://www.cdc.gov/mmwr/volumes/66/wr/mm6630a6.htm. Accessed December 11, 2017.
14. National Alliance on Mental Illness. Depression. http://www.nami.org/Learn-More/Mental-Health-Conditions/Depression. Accessed January 3, 2018.
15. Khoddam R, Jackson NJ, Leventhal AM. Internalizing symptoms and conduct problems: Redundant, incremental, or interactive risk factors for adolescent substance use during the first year of high school? *Drug Alcohol Depend.* 2016;169:48-55.
16. Fergusson DM, Woodward LJ. Mental health, educational, and social role outcomes of adolescents with depression. *Arch Gen Psychiat.* 2002;59(3):225-231.
17. Thapar A, Collishaw S, Pine DS, Thapar AK. Depression in adolescence. *Lancet.* 2012;379(9820):1056-1067.
18. Thomas JF, Temple JR, Perez N, Rupp R. Ethnic and gender disparities in needed adolescent mental health care. *J Health Care Poor Underserved.* 2011;22(1):101-110.

19. Lau M, Hua L, Flores G. Racial/ethnic disparities in health and health care among US adolescents. *Health Serv Res*. 2012;47(5):2031-2059.
20. Irwin CE, Jr., Adams SH, Park MJ, Newacheck PW. Preventive care for adolescents: few get visits and fewer get services. *Pediatrics.* 2009;123(4):e565-572.
21. Dixon SK, Hoopes AJ, Benkeser D, Grigg A, Grow HM. Characterizing key components of a medical home among rural adolescents. *Journal Adolesc Health.* 2016;58(2):141-147.

Summary of Changes

IRB Approvals				
Study ID	Type of Submission	Date submitted	Date approved	Content of Submission
STUDY00010090	Study created	6/19/2018	8/27/2018	
MOD00016541	Modification	9/19/2018	10/10/2018	Modification request from IRB to address the question of Data Transfer.
MOD00016962	Modification	10/22/2018	12/12/2018	Protocol updates to include the addition of PCORI funding, as well as updates to study aims to differentiate between HRSA and PCORI funding. Language has been updated to match what has been submitted to ClinicalTrials.gov. Also added our Clinicaltrials.gov number to the protocol. Additionally, the PCORI specific opt-out letters (English and Spanish) have been included in this modification. Modifications have also been made to the previously approved HRSA opt-out letters (English and Spanish) to allow for flexibility of return date by each high school. A Letter of Support from [redacted] High School, who participated in the first round of screenings has also been uploaded.
MOD00018762	Modification	3/26/2019	4/11/2019	Protocol updates to sections 3-7 to include language regarding Feedback Guides, created/uploaded the Feedback Guide as a supporting document, added [redacted] to the study team (as well as updated the Study Team Qualifications document).
MOD00019992	Modification	7/1/2019	7/11/2019	Protocol updated to include all participating schools, as well as letters of support.
MOD00020599	Modification	8/15/2019	8/23/2019	Added study team members [redacted] and [redacted]. Removed [redacted] as a study team member.<br><br>Updated HRP-591 Protocol- Sections 1.0, 3.0, 4.0 and 8.0 (updates relate to the total number of schools, total anticipated sample size based on participating schools).
MOD00020855	Modification	9/3/2019	9/9/2019	Updated the first phone number for questions/concerns for the study team to the following documents: PCORI Opt-Out Letter (Spanish), PCORI Opt-Out Letter (English), HRSA Opt-out Letter (Spanish), and HRSA Opt-out Letter English.
MOD00021609	Modification	10/30/2019	11/14/2019	Added study team member [redacted].
RNI00005068	Reportable New Information	11/26/2019	12/2/2019	Submitted RNI to notify IRB that [redacted] has opted to not continue with the study.
MOD00022346	Modification	1/9/2020	NA	DISCARDED TO ADD UPDATES TO STUDY TEAM. Updated HRP-591 Protocol sections 1.0, 3.0, 4.0, and 8.0 that [redacted] is no longer participating. Updated study related numbers to reflect this change.

MOD00022348	Modification	1/9/2020	2/13/2020	Updated HRP-591 Protocol sections 1.0, 3.0, 4.0, and 8.0 that [redacted] is no longer participating. Updated study related numbers to reflect this change. [redacted] was added as a study team member and HRP-509 was updated.
MODCR0004732`	Modification/CR	3/24/2020	4/9/2020	Continuing review submitted; modifications made to HRP-591 Protocol to remove [redacted] as a participant; [redacted]'s LOS was marked as INACTIVE.
MOD00023506	Modification	4/9/2020	4/9/2020	Removed [redacted] as a team member, updates made to HRP-509.
MOD00023579	Modification	4/14/2020	4/17/2020	Updates made to Feedback Guides to include questions relating to COVID 19. Year 2 Feedback Guides were also added to supporting documents. Edits made to HRP-591.
MOD00023958	Modification	5/15/2020	6/5/2020	Added [redacted] as a team member; updates to HRP-591 sections 4.2 and 4.3 to include verbiage re: [redacted]'s opt-out letter. Issue was that this school did not send opt-out letters to parents whose students were randomized to the target (control) arm of the trial. An updated letter was included in supporting documents to be sent in spring 2020, after the intervention ended. No information will be collected for those students whose parents return these opt-out forms by the end of the school year. Also, intervention timeline extensions (Sept-Mar), and intervention/data collection updates due to COVID-19 were added.
RNI00005568/RNI00005583	Reportable New Information	5/15/2020<br>5/19/2020	Discarded	Corresponding RNI to MOD00023958, included signed report from DSMB meeting on May 11, 2020. Discarded on 5/19, per IRB Request. IRB contacted team on 5/15 to confirm that this should not have been discarded because it was a protocol deviation. Resubmitted on 5/15 as, RNI00005583: Protocol Deviation and Protocol/Intervention Updates.
RNI00006159	Reportable New Information	1/12/2021	3/22/2021	Per our protocol, every student at a participating school receives a unique study identification number, ensuring any student whose parents have opted-out are not tracked throughout the year. In turn, this safeguards against any student whose parents opt-out from participating in the screening process. While comparing screening data (saved in REDCap) and completed school spreadsheets, the study team realized 4 students at [redacted] High School whose parents had provided opt-out letters did participate in the mental health screener. Our biostatistician was made aware of the situation ahead of analysis and this matter was added to our DSMB meeting agenda on February 11, 2021. A supporting letter from the DSMB indicating the study could continue as planned was added to this RNI.
MODCR00005258	Modification/CR	1/27/2021	2/8/2021	Continuing review submitted; modifications to update team member profile.
