# Supplementary material for: Screening in High Schools to Identify, Evaluate, and Lower Depression Among Adolescents: A Randomized Clinical Trial
Source: JAMA Netw Open. 2021 Nov 5;4(11):e2131836. doi: 10.1001/jamanetworkopen.2021.31836 (PMC8571659; doi:10.1001/jamanetworkopen.2021.31836)
Supplement: Supplement 2. — eTable 1. Definitions of Each Criterion That Comprise the Primary Outcome eTable 2. Reasons Students Did Not Meet Criterion 2 (Confirmed) Among Those Who Met Criterion 1 (Identified) eTable 3. Reasons Students Did Not Meet Criterion 3 (Initiated) Among Those Students Who Met Criterion 2 (Confirmed) eTable 4. Percentage of Students With Each Outcome by Subgroups of Sex, Race/Ethnicity, and School Location [file jamanetwopen-e2131836-s002.pdf]

Supplementary Online Content

Sekhar DL, Schaefer EW, Waxmonsky JG, et al. Screening in high schools to identify, evaluate, and lower depression among adolescents: a randomized clinical trial. *JAMA Netw Open*. 2021;4(11):e2131836. doi:10.1001/jamanetworkopen.2021.31836

eTable 1. Definitions of Each Criterion That Comprise the Primary Outcome

eTable 2. Reasons Students Did Not Meet Criterion 2 (Confirmed) Among Those Who Met Criterion 1 (Identified)

eTable 3. Reasons Students Did Not Meet Criterion 3 (Initiated) Among Those Students Who Met Criterion 2 (Confirmed)

eTable 4. Percentage of Students With Each Outcome by Subgroups of Sex, Race/Ethnicity, and School Location

This supplementary material has been provided by the authors to give readers additional information about their work.

© 2021 Sekhar DL et al. *JAMA Network Open.*

**eTable 1**. Definitions of Each Criterion That Comprise the Primary Outcome	
**Criterion**	**Definition**
Identified	Patient Health Questionnaire-9 (PHQ-9) score >10 or<br>PHQ-9 question 9 score ≥1 or<br>Student Assistance Program (SAP) referral with one of the following primary or secondary referral reasons:<br>• Behavioral concern<br>• Externalizing behaviors<br>• Internalizing behaviors<br>• Self-harm<br>• Suicidal ideation<br>• Other – if depressed/depression or "past thoughts of harming self" specified<br>• Other – if PHQ-9 screening specified
Confirmed	A SAP referral with a response of "yes" to the question "Was SAP warranted?" or<br>Missing response to that question but recommended services indicated. SAP does not diagnose but will consider the student's individual situation via additional probing and make a recommendation accordingly.<br>Note: For students who met the identified criterion due to PHQ-9 result, a further requirement was a SAP referral that had the same primary and secondary referral reasons listed above.
Initiated	Received one of the following primary or secondary school-based, community-based, or liaison services recommended by the SAP team:<br>• Behavioral<br>• Crisis intervention<br>• Family-Based Mental Health Services or Behavioral Health Rehabilitation Services<br>• In-school support groups<br>• Mental health assessment<br>• Mental health treatment<br>• One-to-one counseling with guidance counselor, school psychologist, etc.<br>• One-to-one follow-up with team member<br>• One-to-one follow-up with team member or other school personnel<br>• One-to-one with mental health liaison<br>• Other mental health treatment<br>• Other support services<br>• Outpatient therapy<br>• Psychiatric inpatient<br>• Psychiatric outpatient<br>• Referral for further mental health assessment<br>• School based mental health group<br>• Screening by behavioral health SAP liaison

© 2021 Sekhar DL et al. *JAMA Network Open.*

	• Screening by mental health SAP liaison<br>• Screening with mental health liaison<br>• Services by/from school social worker<br>• Therapy

© 2021 Sekhar DL et al. *JAMA Network Open.*

**eTable 2.** Reasons Students Did Not Meet Criterion 2 (Confirmed)[a] Among Those Who Met Criterion 1 (Identified)			
	Targeted screening (N=136)	Universal screening (N=793)	Total (N=929)
**Reasons**			
Student Assistance Program (SAP) referral not warranted	3 (2.2%)	83 (10.5%)	86 (9.3%)
Already in services/treatment	0 (0.0%)	30 (3.8%)	30 (3.2%)
Referred to outside agency, no further information	42 (30.9%)	156 (19.7%)	198 (21.3%)
Met with (or referred to) counselor, no further SAP engagement	24 (17.6%)	205 (25.9%)	229 (24.7%)
Met with school social worker (suicide liaison), no further SAP engagement	38 (27.9%)	39 (4.9%)	77 (8.3%)
Met with school staff (role not specified in data provided by school), no further SAP engagement	28 (20.6%)	152 (19.2%)	180 (19.4%)
Parent/student refusal or no parental response	1 (0.7%)	38 (4.8%)	39 (4.2%)
Parent will seek outside resources	0 (0.0%)	12 (1.5%)	12 (1.3%)
Unenrolled/transferred	0 (0.0%)	6 (0.8%)	6 (0.6%)
Recommended services, no further information	0 (0.0%)	15 (1.9%)	15 (1.6%)
No information provided	0 (0.0%)	56 (7.1%)	56 (6%)
Referred to crisis, no further SAP engagement	0 (0.0%)	1 (0.1%)	1 (0.1%)

[a]identified (major depressive disorder [MDD] symptoms): positive Patient Health Questionnaire-9 (score >10 or response ≥1 on question 9) or behavior prompting referral to SAP for MDD; confirmed: SAP does not diagnose, but to meet criteria, SAP must confirm identified behavior/symptoms warrant further evaluation

© 2021 Sekhar DL et al. *JAMA Network Open.*

**eTable 3.** Reasons Students Did Not Meet Criterion 3 (Initiated)[a] Among Those Students Who Met Criterion 2 (Confirmed)			
	Targeted screening (N=29)	Universal screening (N=151)	Total (N=180)
**Reasons**			
Parent refusal/written permission not obtained	14 (48.3%)	67 (44.4%)	81 (45%)
Student refusal	3 (10.3%)	17 (11.3%)	20 (11.1%)
Student already in treatment	4 (13.8%)	57 (37.7%)	61 (33.9%)
Did not receive recommended services	1 (3.4%)	1 (0.7%)	2 (1.1%)
Other	7 (24.1%)	9 (6.0%)	16 (8.9%)

[a]confirmed: Student Assistance Program (SAP) does not diagnose, but to meet criteria, SAP must confirm identified behavior/symptoms warrant further evaluation; initiated: participated in at least one SAP recommended treatment/service for major depressive disorder, e.g., follow-up mental health supports

© 2021 Sekhar DL et al. *JAMA Network Open.*

**eTable 4.** Percentage of Students With Each Outcome by Subgroups of Sex, Race/Ethnicity, and School Location				
**Subgroup**	**Outcome**		**Targeted screening**	**Universal screening**
**Sex**	Totals	Male	3495 (100%)	3468 (100%)
		Female	2941 (100%)	3005 (100%)
	Identified[a]	Male	110 (3.1%)	400 (11.5%)
		Female	90 (3.1%)	626 (20.8%)
	Confirmed	Male	33 (0.9%)	80 (2.3%)
		Female	31 (1.1%)	153 (5.1%)
	Initiated[b]	Male	15 (0.4%)	28 (0.8%)
		Female	20 (0.7%)	52 (1.7%)
**Race/ethnicity**	Totals	Non-Hispanic white	2857 (100%)	2985 (100%)
		Non-Hispanic Black	1571 (100%)	1320 (100%)
		Hispanic	1266 (100%)	1421 (100%)
		Other	742 (100%)	747 (100%)
	Identified	Non-Hispanic white	61 (2.1%)	474 (15.9%)
		Non-Hispanic Black	95 (6.0%)	187 (14.2%)
		Hispanic	28 (2.2%)	204 (14.4%)
		Other	16 (2.2%)	161 (21.6%)
	Confirmed	Non-Hispanic white	48 (1.7%)	115 (3.9%)
		Non-Hispanic Black	10 (0.6%)	37 (2.8%)
		Hispanic	5 (0.4%)	67 (4.7%)
		Other	1 (0.1%)	14 (1.9%)
	Initiated[b]	Non-Hispanic white	27 (0.9%)	42 (1.4%)
		Non-Hispanic Black	6 (0.4%)	13 (1.0%)
		Hispanic	2 (0.2%)	16 (1.1%)
		Other	0 (0.0%)	9 (1.2%)
**Location**	Totals	Urban	5728 (100%)	5735 (100%)
		Rural	708 (100%)	738 (100%)
	Identified	Urban	189 (3.3%)	893 (15.6%)
		Rural	11 (1.6%)	133 (18.0%)
	Confirmed	Urban	55 (1.0%)	212 (3.7%)
		Rural	9 (1.3%)	21 (2.8%)
	Initiated[b]	Urban	28 (0.5%)	69 (1.2%)
		Rural	7 (1.0%)	11 (1.5%)

[a]identified (major depressive disorder [MDD] symptoms): positive Patient Health Questionnaire-9 (score >10 or response ≥1 on question 9) or behavior prompting referral to Student Assistance Program (SAP) for MDD; confirmed: SAP does not diagnose, but to meet criteria, SAP must confirm identified behavior/symptoms warrant further evaluation; initiated: participated in at least one SAP recommended treatment/service for MDD, e.g., follow-up mental health supports.
[b]two students had missing values

© 2021 Sekhar DL et al. *JAMA Network Open.*
