# Supplementary material for: Screening in High Schools to Identify, Evaluate, and Lower Depression Among Adolescents: A Randomized Clinical Trial
Source: JAMA Netw Open. 2021 Nov 5;4(11):e2131836. doi: 10.1001/jamanetworkopen.2021.31836 (PMC8571659; doi:10.1001/jamanetworkopen.2021.31836)
Supplement: Supplement 3. — Data Sharing Statement [file jamanetwopen-e2131836-s003.pdf]

Data Sharing Statement

Sekhar. Screening in High Schools to Identify, Evaluate, and Lower Depression Among Adolescents. *JAMA Netw Open*. Published November 05, 2021. doi:10.1001/jamanetworkopen.2021.31836

Data
Data available: Yes
Data types: Deidentified participant data, Data dictionary
How to access data: dsekhar@pennstatehealth.psu.edu
When available: beginning date: 12-01-2022

Supporting Documents
Document types: None

Additional Information
Who can access the data: Researchers whose proposed use of the data has been approved.
Types of analyses: For any purpose.
Mechanisms of data availability: After proposed use of data is approved.
